# Reactive metal boride nanoparticles trap lipopolysaccharide and peptidoglycan for bacteria-infected wound healing

**DOI:** 10.1038/s41467-022-35050-6

**Published:** 2022-11-29

**Authors:** Yun Meng, Lijie Chen, Yang Chen, Jieyun Shi, Zheng Zhang, Yiwen Wang, Fan Wu, Xingwu Jiang, Wei Yang, Li Zhang, Chaochao Wang, Xianfu Meng, Yelin Wu, Wenbo Bu

**Affiliations:** 1 Tongji University Cancer Center, Shanghai Tenth People’s Hospital, Tongji University School of Medicine, Shanghai, 200072 P. R. China; 2 Department of Materials Science and State Key Laboratory of Molecular Engineering of Polymers, Fudan University, Shanghai, 200433 P. R. China; 3 School of Life Sciences and Technology, Tongji University, Shanghai, 200092 P. R. China; 4 School of Life Science, East China Normal University, Shanghai, 200241 P. R. China

**Keywords:** Biomaterials, Nanobiotechnology, Biomedical materials

## Abstract

Bacteria and excessive inflammation are two main factors causing non-healing wounds. However, current studies have mainly focused on the inhibition of bacteria survival for wound healing while ignoring the excessive inflammation induced by dead bacteria-released lipopolysaccharide (LPS) or peptidoglycan (PGN). Herein, a boron-trapping strategy has been proposed to prevent both infection and excessive inflammation by synthesizing a class of reactive metal boride nanoparticles (MB NPs). Our results show that the MB NPs are gradually hydrolyzed to generate boron dihydroxy groups and metal cations while generating a local alkaline microenvironment. This microenvironment greatly enhances boron dihydroxy groups to trap LPS or PGN through an esterification reaction, which not only enhances metal cation-induced bacterial death but also inhibits dead bacteria-induced excessive inflammation both in vitro and in vivo, finally accelerating wound healing. Taken together, this boron-trapping strategy provides an approach to the treatment of bacterial infection and the accompanying inflammation.

## Abstract (web summary)

Antibacterial wound healing approaches often target bacteria but overlook the inflammation response caused by products release by dead bacteria. Here, the authors report on the development of Boride nanoparticles to treat infection and prevent excessive inflammation by trapping lipopolysaccharides/peptidoglycans.

## Introduction

Impaired wound healing is a growing global concern driven by aging populations and the increasing prevalence of chronic conditions, such as diabetes and obesity. It affects ∼20 million individuals worldwide and over $31 billion has been spent annually on their treatment and management^1^. Infection is a major factor that delays wound healing, which can lead to sepsis and multiorgan failure, and even cause death in severe cases^2,3^. Currently, antibiotics are the main strategies to reduce the bacterial burden for wound healing and lower the mortality rates after severe infection. However, over-prescription and misuse of antibiotics have contributed to the emergence of antibiotic-resistant organisms, limiting the treatment efficacy of wound healing^4^. Moreover, the dead bacteria accumulating at the site of infection could induce undesirable tissue inflammation^5,6^. One of the most important reasons is that the dead bacteria can release massive amounts of endotoxin (lipopolysaccharide (LPS) in Gram-negative bacteria and peptidoglycan (PGN) in Gram-positive bacteria) that can activate immune cells to induce excessive inflammation, leading to impaired wound healing, systemic inflammation, intravascular coagulation, and organ dysfunction^7–9^. This is a leading cause of death in the US alone, with over 700,000 cases estimated every year, and the mortality rate ranges from 28% to 60%^10^. Therefore, developing strategies to simultaneously inhibit the survival of live bacteria and dead bacteria-induced excessive inflammation to heal infected wounds is urgently needed.

Certain components of pathogens are crucial for their structure and function, ensuring pathogen survival and pathogenicity. LPS and PGN are typically the key components of Gram-negative and Gram-positive bacteria, respectively^11^. On one hand, LPS/PGN is a structural component of the bacteria cell wall, which maintains the integrity of the bacteria and protects the bacteria against antibacterial treatments^12^. On the other hand, LPS/PGN is the main functional component of the endotoxin, which is released from the bacterial surface upon bacteria die or lyse. These free LPS/PGN can induce excessive inflammation and toxicity to the host^13,14^. Therefore, targeting the key component of bacteria would structurally inhibit bacterial survival and functionally suppress dead bacteria-induced excessive inflammation and toxicity. Currently, LPS-binding peptides have been reported to react with LPS, resulting in antibacterial activity or the inhibition of LPS/PGN-induced immune cell activation^10,15,16^, however, the precise mechanism by which these peptides perform their biological activities remains elusive. Furthermore, the poor bioavailability and the proteolytic stability of peptides limit their application in clinic^17^. Therefore, targeting the key component of bacteria (LPS/PGN) to simultaneously inhibit bacterial survival and dead bacteria-induced excessive inflammation for wound healing is still facing serious challenges.

LPS/PGN is mainly composed of many different sugars such as hexose or pentose, which contains many 1,2 or 1,3-dial dihydroxyl groups^18,19^. Studies have shown that borate materials can produce dynamic borate ester bonds through an esterification reaction with the dihydroxyl groups^20^. This dynamic covalent bond has been widely used to identify substances such as blood, glucose, and ATP^21–23^. Considering that bacterial LPS/PGN contains structures of 1,2-diol or 1,3-diol and that borate derivatives are prone to react with diol-containing compounds to form borate-diol esters^24,25^, materials possessing boron dihydroxyl functional groups may react with LPS/PGN to inhibit bacterial survival and simultaneously reduce dead bacteria-induced inflammation. However, such dynamic covalent bonds easily dissociate under acidic and inflammatory conditions. Hence, it is necessary to design an antibacterial reagent that efficiently forms stable borate ester bonds with the key component of bacterial LPS/PGN, finally promoting wound healing.

Reactive metal borides (MB NPs) have rich bonding characteristics, among which the strength of B–M ion bond is relatively weak. Therefore, MB NPs undergo the ion exchange reactions with protons during hydrolysis, and the breaking of boron–metal ion bonds, leading to the release of hydroxide, cation ions, and the formation of boron hydride (BH) nanosheets^26^. In addition, BH nanosheets can be further hydrolyzed to generate boron dihydroxyl groups. Meanwhile, the hydroxides released by the MB NPs can regulate the configuration transition of boron atom, which is helpful for the complexation of the boron dihydroxyl groups with LPS/PGN to form more stable O–B–O bonds. Therefore, MB NPs are the ideal material system to trap LPS/PGN. Moreover, the strength of B–M ion bond of the corresponding MB NPs varies greatly with the selection of different raw metal materials. Taking Mg, Al, and Be as examples, after the reaction of these small-sized, low-charge metal elements with boron, the corresponding B–M bonds are significantly reduced^27^, which effectively improve the hydrolysis ability of the MB NPs, accelerating the generation of boron dihydroxyl groups, and facilitating the complex reaction of such material system with LPS/PGN. Since metal cations such as silver^23^, magnesium^28^, aluminum^29^, bismuth^30,31^, cobalt^32^, among others^33–35^ are often responsible for antibacterial activities and the B–M bond energy can be affected by the metal cations, the selection of metal components is very important to regulate the hydrolysis ability of this material system. Therefore, we hypothesized that MB NPs (M = Mg, Al, and Be) could form stable borate ester bonds with LPS/PGN, the key component of bacteria, consequently inhibiting bacterial survival and decreasing dead bacteria-induced excessive inflammation for wound healing.

**Fig. 1:**
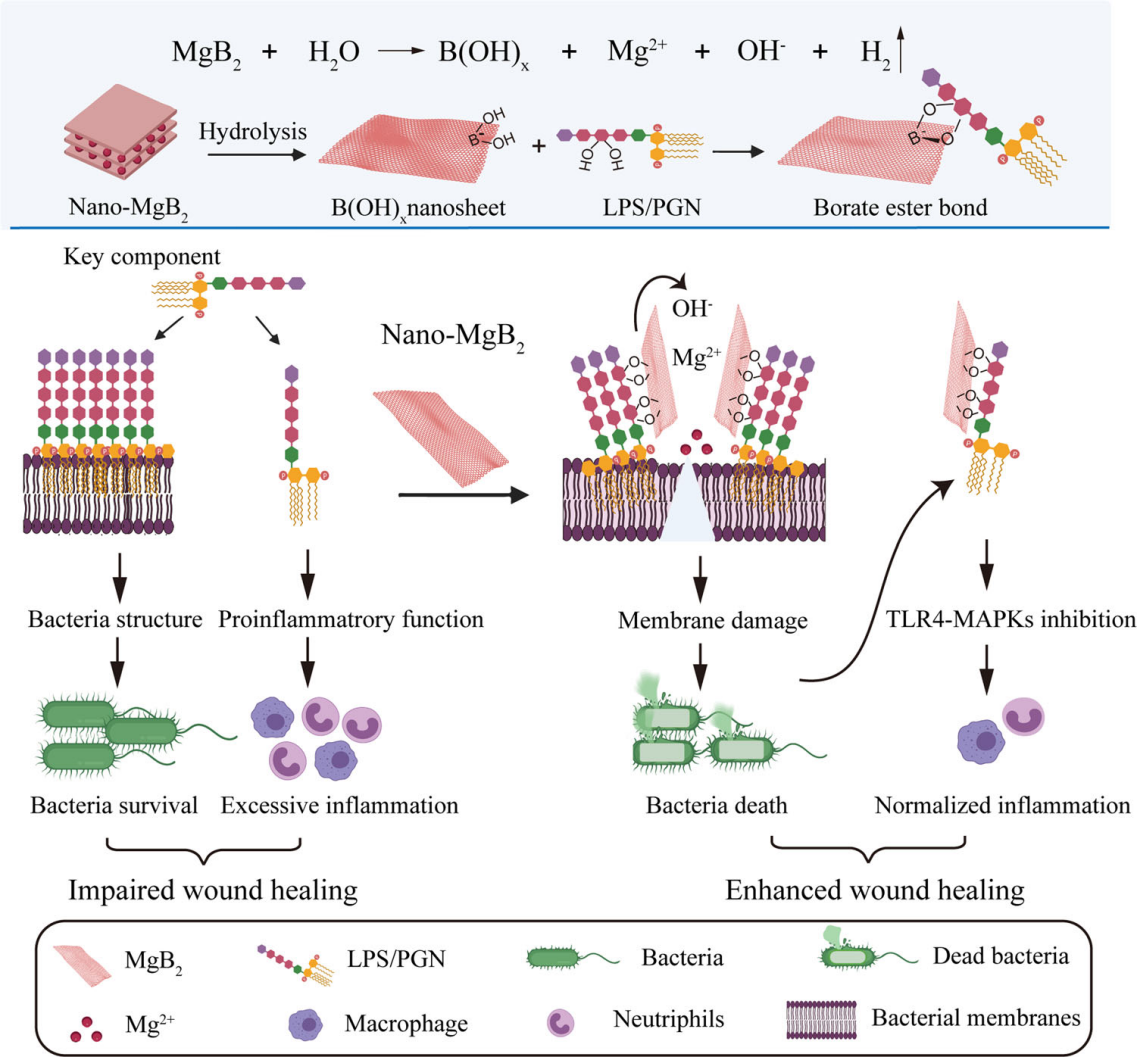
Boron-trapping strategy for bacteria-infected wound healing. Reactive metal borides (such as Nano-MgB_2_) were gradually hydrolyzed to generate boron dihydroxy groups (HO–B–OH) and metal cations (Mg^2+^) while generating a local alkaline microenvironment. The alkaline microenvironment promoted the HO–B–OH to trap the key component of bacteria (LPS/PGN), which not only inhibited the survival of live bacteria, but also block the excessive inflammatory response of immune cells induced by dead bacteria-released LPS/PGN, resulting in enhanced wound healing. This figure is created with BioRender.com.

Herein, we proposed a boron-trapping strategy and synthesized a class of nanoscale MB NPs (M = Mg, Al, and Be), using Nano-MgB_2_ as a representative example to elucidate the mechanism and function of MB NPs in promoting infected wound healing (Fig. 1). The Nano-MgB_2_ could gradually hydrolyze to generate boron dihydroxy groups and metal cations while generating a local alkaline/low reactive oxygen species (ROS) microenvironment. The alkaline microenvironment could promote the Nano-MgB_2_ to trap much more LPS/PGN through the esterification reaction between the boron dihydroxyl group and diol of LPS/PGN. This process could not only induce a high focal concentration of Mg^2+^ on the bacterial membrane, enhancing the ability of Mg^2+^ to disrupt the membrane structure of living bacteria, but also trap dead bacteria-released LPS/PGN from escaping, resulting in the inhibition of LPS/PGN-induced excessive inflammation. The suppression of both bacterial growth and excessive inflammation could significantly promote wound healing. This boron-trapping strategy can be used to develop methods for promoting the healing of infected wounds.

## Results

### Synthesis and characterization of Nano-MgB_2_

**Fig. 2:**
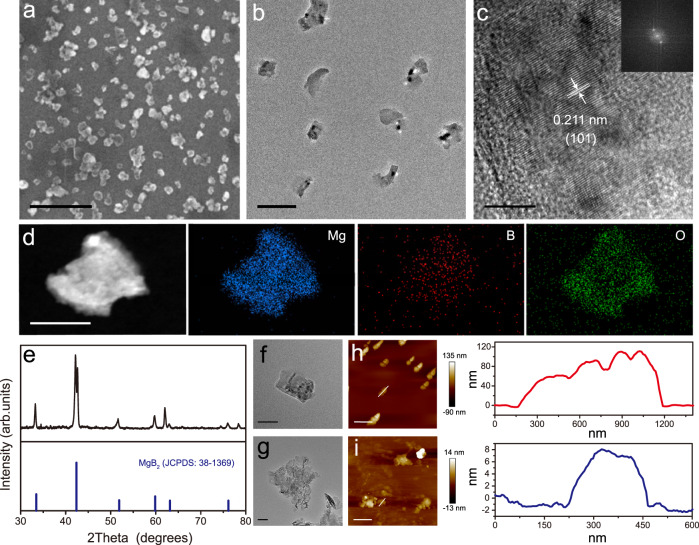
Morphology and characterization of Nano-MgB_2_. **a** SEM image of Nano-MgB_2_. Scale bar = 500 nm. **b** TEM image of Nano-MgB_2_. Scale bar = 200 nm. **c** HRTEM image of Nano-MgB_2_. Scale bar = 5 nm. Inset graph: fast Fourier transform image of Nano-MgB_2_. **d** HADDF-STEM and EDS elemental mapping images of Nano-MgB_2_. Scale bar = 100 nm. **e** XRD patterns of Nano-MgB_2_. **f**, **g** TEM images of a single particle of Nano-MgB_2_ before and after hydrolysis. Scale bar = 50 nm. **h**, **i** AFM images of Nano-MgB_2_ before and after hydrolysis. Scale bar (up) = 1 μm, scale bar (down) = 500 nm. Data are representative of at least three independent experiments with similar results. Source data are provided as a Source data file.

As proof of concept, MB NPs, a series of boron-trapping materials, were synthesized using an improved self-propagating high-temperature synthesis (SHS) approach^36^. As seen in Supplementary Fig. 1 as well as Fig. 2e, the X-ray diffraction (XRD) results demonstrated that Nano-MgB_2_ can be indexed to the hexagonal phase of MgB_2_ (JCPDS: 38-1369), Nano-AlB_2_ can be indexed to the hexagonal phase of AlB_2_ (JCPDS: 39-1483), and Nano-BeB_x_ can be ascribed to the mixture of the tetragonal phase of BeB_6_ (JCPDS: 13-0361) and hexagonal phase of BeB_12_ (JCPDS: 19-0152). Dynamic light scattering (DLS) results revealed that the mean diameter of MB NPs was about 150–250 nm (Supplementary Fig. 2). We hypothesized that these three MB NPs had antibacterial effects. Therefore, we incubated different concentrations of MB NPs with *Pseudomonas aeruginosa*, the most common Gram-negative bacteria in the chronic wounds^5^, to screen the most effective antibacterial MB NP. As shown in Supplementary Fig. 3, all MB NPs exhibited significant antibacterial effects, however, Nano-MgB_2_ showed a stronger antibacterial effect compared to AlB_2_ and BeB_x_. Therefore, we used Nano-MgB_2_ as a representative example to evaluate the synthesis, characterization, and function of MB NPs.

As shown in Fig. 2a, b, scanning electron microscopic (SEM) and transmission electron microscopic (TEM) images indicated that the obtained Nano-MgB_2_ was well dispersed in H_2_O. High-resolution TEM (HRTEM) analysis clearly revealed the lattice fringes of Nano-MgB_2_ with *d*-spacing of 2.11 Å, corresponding to the crystalline face (101) of Nano-MgB_2_ (Fig. 2c). Moreover, the images obtained using low-magnification high-angle annular dark-field scanning TEM (HAADF-STEM) as well as the elemental mapping indicated that this nanoplatform was mainly composed of Mg, B, and O (Fig. 2d). Because of the layered properties of Nano-MgB_2_, it formed 2D-like nanostructures after hydrolysis (Fig. 2f, g). Moreover, atomic force microscopy (AFM) indicated that the thickness of Nano-MgB_2_ decreased from 60–100 nm before hydrolysis to 7–8 nm after hydrolysis (Fig. 2h, i) further demonstrating that the morphology of Nano-MgB_2_ changed from nanoparticles to 2D nanosheets. All these data demonstrated that the designed nanoparticles were successfully synthesized.

### Functional characterization of Nano-MgB_2_

**Fig. 3:**
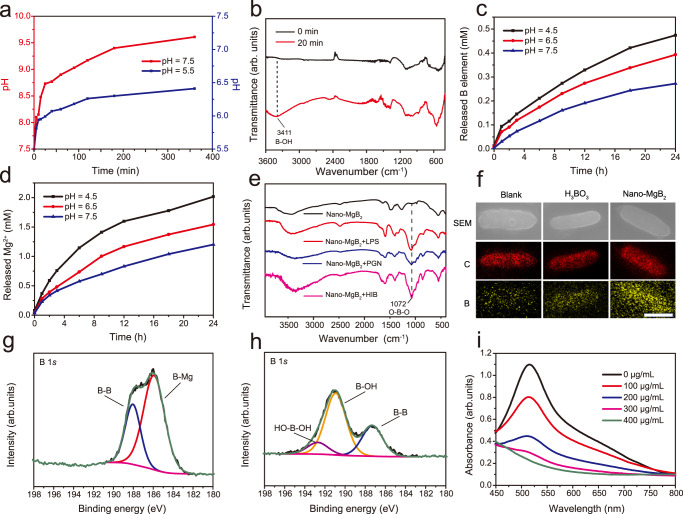
Functional characterization of Nano-MgB_2_. **a** Change of pH over time after Nano-MgB_2_ hydrolysis. **b** FTIR spectra of Nano-MgB_2_ after hydrolysis. **c**, **d** The releasing behaviors of B and Mg elements after Nano-MgB_2_ hydrolysis in buffer solution with different pH. **e** FTIR spectra of O–B–O formation under various conditions. **f** Elemental mapping of *P. aeruginosa* incubated with H_3_BO_3_ and Nano-MgB_2_ for 3 h. Scale bar = 500 nm. C, carbon atom. B, boron atom. **g**, **h** XPS fine spectra of Nano-MgB_2_ before and after hydrolysis. **i** UV-vis spectra of DPPH• scavenging ability with different concentrations of Nano-MgB_2_. Data are representative of at least three independent experiments with similar results. Source data are provided as a Source data file.

To determine the functional characterization of Nano-MgB_2_, we first evaluated its ability to generate a weakly alkaline microenvironment, Mg^2+^, and boron hydroxyl groups. As shown in Fig. 3a, the pH rapidly increased within 30 min and became nearly stable at around 200 min (pH stabilized at 9.5 in a buffer solution of pH = 7.5 and at 8.5 in a buffer solution of pH = 5.5). As shown in FTIR spectrum (Fig. 3b), the characteristic peak of boron hydroxyl groups (broad peak at 3000–3500 cm^−1^) began to appear after about 20 min of Nano-MgB_2_ hydrolysis, which suggested that Nano-MgB_2_ can exhibit an instant therapeutic effect on the minute scale and the produced boron hydroxyl groups can exist stably for at least 15 days (Supplementary Fig. 4). Furthermore, as shown in Fig. 3c, d, buffer solution with a lower pH led to a faster hydrolysis rate of Nano-MgB_2_. These data demonstrate that the hydrolysis of MB NPs leads to the generation of an alkaline microenvironment with metal ions and boron hydroxyl groups. To determine whether boron hydroxyl groups act as a boron-trapping agent to trap LPS/PGN, the key components of Gram-negative and -positive bacteria, Nano-MgB_2_ was incubated with LPS, PGN, or dead *P. aeruginosa* (HIB, heat-inhibited bacteria). As shown in Fig. 3e, the characteristic peak at 1072 cm^−1^ demonstrated that the hydrolysate of Nano-MgB_2_ reacted with LPS, PGN, and HIB to form boronic ester (O–B–O) bonds. Besides Nano-MgB_2_, Nano-AlB_2_ and Nano-BeB_x_ can also react with LPS and PGN to form O–B–O bonds, suggesting that members of this class of nanoscale MB NPs have similar boron-trapping functions (Supplementary Fig. 5). Furthermore, we compared the LPS/PGN binding activity of Nano-MgB_2_ with that of H_3_BO_3_ which contains boron hydroxyl groups. As shown in SEM images equipped with elemental mapping, a much larger amount of B element was enriched on the bacteria in the Nano-MgB_2_-incubated group than that in the H_3_BO_3_-incubated group (Fig. 3f and Supplementary Fig. 6). To analyze this phenomenon, the borate ester bonds formed by the complexation of H_3_BO_3_ and hydrolysate of nano-MgB_2_ with the bacterial polysaccharide fraction were calculated based on density functional theory (DFT). Taking LPS as an example, its polysaccharide component is composed of a unique bicyclic monosaccharide structure, and each sugar motif has two binding sites (3, 4-o-hydroxyl or 4, 5-o-hydroxyl) that can complex with boron dihydroxyl groups. For the binding site of 3, 4-o-hydroxyl (Supplementary Figs. 7–8), the C–O bond lengths of the borate ester bond formed by H_3_BO_3_ (1.451 Å/1.451 Å) are longer than that of nano-MgB_2_ hydrolysate (1.431 Å/1.439 Å), indicating that the C–O bond formed by the former is easier to break. Moreover, we used crystal orbital Hamilton population (COHP) analysis to reflect the strength of C–O bonding. The values of ICOHP of the C–O bonds formed by nano-MgB_2_ hydrolysate (−8.853 eV/−9.071 eV) are much negative compared to H_3_BO_3_ (−8.592 eV/−8.655 eV), which directly proves the enhancement of the C–O bonds. For the binding site of 4, 5-o-hydroxyl, we obtained similar theoretical calculation results (Supplementary Figs. 9–10). The above data indicate that Nano-MgB_2_ forms more stable borate ester bonds with LPS when compared to H_3_BO_3_.

In addition, X-ray photoelectron spectroscopy (XPS) was performed to analyze the metal components of MB NPs (Supplementary Figs. 11–13). According to the survey XPS spectra, O and corresponding metal signals can be clearly observed. Specifically, the Mg 2*p* signal of the Nano-MgB_2_ consisted of Mg^2+^ (50.5 eV) alone, the Al 2*p* signal of the Nano-AlB_2_ consisted of Al^3+^ (74.3 eV) alone, and the Be 1*s* signal of the Nano-BeB_x_consisted of Be^2+^ (113.5 eV) alone. XPS was also utilized to characterize the hydrolysis process of MB NPs. Take Nano-MgB_2_ as an example (Fig. 3g, h), the sample was characterized by the disappearance of the characteristic peak for the negatively charged boron species (B-Mg bond, 185.8 eV) and the appearance of the characteristic peak for the positively charged boron species (190.9 eV for B–OH bond and 192.7 eV for HO–B–OH bond, respectively) after hydrolysis. Therefore, the hydrolysis of Nano-MgB_2_ involved oxidation of boron elements. Considering the oxidative stress microenvironment of the bacteria-infected wound, we further examined the ROS-scavenging ability of Nano-MgB_2_. Nano-MgB_2_ exhibited high reactivity towards broad-spectrum ROS (Fig. 3i), reactive nitrogen species (RNS) (Supplementary Fig. 14), and the direct •OH scavenging ability of Nano-MgB_2_ (Supplementary Fig. 15). Thus, the designed MB NPs are gradually hydrolyzed to generate boron dihydroxy groups and metal cations while generating a local alkaline/low ROS microenvironment, which promoted the esterification reaction between boron hydroxyl groups and LPS/PGN.

### Nano-MgB_2_ disrupted the structure of bacteria in vitro

**Fig. 4:**
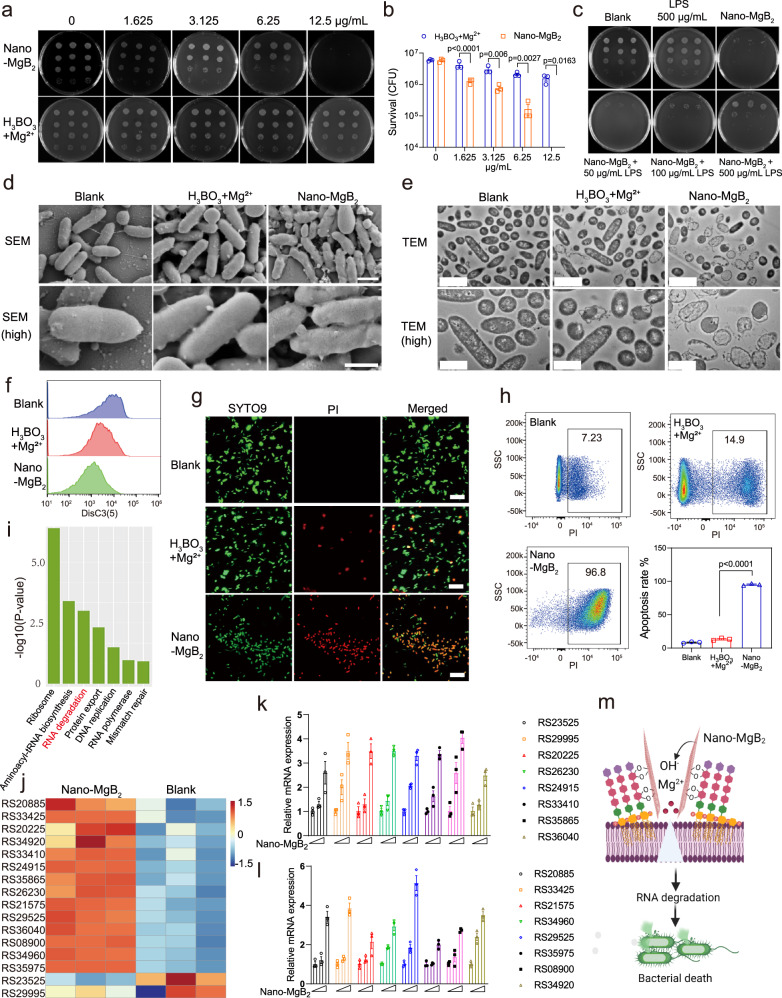
Nano-MgB_2_ reacted with LPS to disrupt bacterial cell membrane. **a** *P. aeruginosa* treated with different concentrations of Nano-MgB_2_ using colony-forming units counting method. **b** Survival rates of *P. aeruginosa* taken as in (**a**) (*n* = 3 biological independent cells). **c** Bacterial survival after *P. aeruginosa* treated with Nano-MgB_2_ in the presence of different concentrations of LPS. **d** SEM images of *P. aeruginosa* cells subjected to Nano-MgB_2_ and H_3_BO_3_ + Mg^2+^. Scale bar (up) = 1 μm, scale bar (down) = 500 nm. **e** TEM images of *P. aeruginosa* cells subjected to Nano-MgB_2_ and H_3_BO_3_ + Mg^2+^. Scale bar (up) = 2 μm, scale bar (down) = 1 μm. **f** Membrane potential of *P. aeruginosa* treated with Nano-MgB_2_ and H_3_BO_3_ + Mg^2+^. **g** Cell membrane permeability of *P. aeruginosa* treated with Nano-MgB_2_ and H_3_BO_3_ + Mg^2+^ by SYTO9 and PI staining. All cells were labeled by the membrane-permeable SYTO9 (green), whereas only cell with damaged membrane were positive for PI (red). Scale bar = 20 μm. **h** FACS analysis of PI-positive bacteria (*n* = 3 biological independent cells). **i** KEGG-pathway of RNA-seq after *P. aeruginosa* treated with Nano-MgB_2_. The *P* values were calculated using hypergeometric distribution. **j** Heatmap of RNA degradation-related gene expression after *P. aeruginosa* treated with Nano-MgB_2_ (gene names are labeled in Supplementary Fig. 18). **k**, **l** QPCR of RNA degradation-related gene expression (*n* = 3 biological independent cells). **m** Schematic illustration of Nano-MgB_2_ reacted with LPS to disrupt cell membrane. Data are representative of at least three independent experiments with similar results. Values are the mean ± SEM. Two-way ANOVA with Bonferroni post test was used in (**b**) and one-way ANOVA with Bonferroni post test was used in (**h**) to analyze multiple groups. Source data are provided as a Source data file.

As the experiments described above demonstrated that Nano-MgB_2_ can react with LPS to form O–B–O bonds, the antibacterial activity of Nano-MgB_2_ was further evaluated. As shown in Fig. 4a, b, Nano-MgB_2_ exhibited excellent antibacterial activity at a low concentration at around 12.5 μg/mL, whereas H_3_BO_3_ + Mg^2+^ group exhibited little or no antibacterial effect against *P. aeruginosa* at the same concentration. This antibacterial effect of our synthesized Nano-MgB_2_ was dramatically stronger than that of commercial MgB_2_ powders^37^, and was comparable to the effects of gentamicin, amikacin, ciprofloxacin, levofloxacin, and better than imipenem, meropenem, ceftazidime, cefepime, ampicillin+sulbactam, aztreonam (Supplementary Fig. 16a). Furthermore, as shown in Supplementary Fig. 17a, b, Nano-MgB_2_ also showed a significant antibacterial effect against *S. aureus*. We also compared the antibacterial activity of Nano-MgB_2_ with three kinds of antibiotics for *S. aureus*. As shown in Supplementary Fig. 16b, the antibacterial effect of Nano-MgB_2_ was better than that of cephalexin, but not that of erythromycin and mupirocin. Taken together, all these data demonstrate that Nano-MgB_2_ has an excellent antimicrobial function which is comparable to the effects of certain kinds of antibiotics.

To further investigate Nano-MgB_2_ trapping LPS is required for the antibacterial effect of Nano-MgB_2_, different concentrations of LPS were added to block the reaction between Nano-MgB_2_ and LPS. Nano-MgB_2_ (6.25 μg/mL) significantly inhibited the growth of *P. aeruginosa*, however, this inhibition effect was rescued by adding LPS, demonstrating that the O–B–O bond between Nano-MgB_2_ and LPS is involved in Nano-MgB_2_-induced bacterial death (Fig. 4c). Furthermore, as shown in the result of SEM of *P. aeruginosa* and *S. aureus*, the bacteria typically had a rod-shaped morphology with a smooth and intact cell wall in the blank and H_3_BO_3_ + Mg^2+^ groups, whereas those treated with Nano-MgB_2_ became wrinkled and shrunk (Fig. 4d and Supplementary Fig. 17c). TEM analysis revealed that the cytoplasm of bacteria was lost in the Nano-MgB_2_-treated group compared with that in the blank and H_3_BO_3_ + Mg^2+^ groups, indicating that degradation of the cytoplasm components or cell membrane leakage occurred after Nano-MgB_2_ treatment (Fig. 4e and Supplementary Fig. 17d). These data demonstrate that Nano-MgB_2_ triggers damage to the bacterial membrane.

Metal cations are often responsible for disrupting the bacterial membrane by altering the membrane potential and permeability. The reaction between MB and LPS on the cell wall not only produces a focal source of metal ions but also narrows the distance between metal cations and cell membrane^38^. Therefore, we first measured the change of the membrane potential in bacteria treated with Nano-MgB_2_ and H_3_BO_3_ + Mg^2+^ using the fluorophore dye DiSC_3_(5) (a cyanine dye that shows increased fluorescence upon dissipation of the membrane potential)^39^. As shown in Fig. 4f, Nano-MgB_2_ significantly changed the membrane potential compared to the blank and H_3_BO_3_ + Mg^2+^ groups, indicating that a focal source of Mg^2+^ from Nano-MgB_2_ damages the cell membrane by changing the membrane potential. Next, we detected the permeability of bacterial membranes after Nano-MgB_2_ treatment. *P. aeruginosa* and *S. aureus* treated with H_3_BO_3_ + Mg^2+^ and Nano-MgB_2_ were stained with SYTO9 and propidium iodide (PI). SYTO9 is a green-fluorescent dye that freely permeates cell membranes and shows a large increase in fluorescence upon binding to nucleic acids. PI is a red-fluorescent dye that can specifically bind to DNA or RNA to enhance fluorescence but cannot pass through the intact membranes of viable cells^39^. As shown in Fig. 4g and Supplementary Fig. 17e, Nano-MgB_2_-treated group exhibited a large number of bacteria with red fluorescence (PI staining), compared with the blank and H_3_BO_3_ + Mg^2+^ groups, suggesting that Nano-MgB_2_ significantly increased the membrane permeability of bacteria. Next, we stained the bacteria with PI and performed fluorescence-activated cell sorting (FACS) to further quantify permeability changes in bacteria in the presence of Nano-MgB_2_. As shown in Fig. 4h and Supplementary Fig. 17f, Nano-MgB_2_ induced a dramatical change in membrane permeability (96.8% in *P. aeruginosa* and 89.0% in *S. aureus*) than that of H_3_BO_3_ + Mg^2+^ (14.9% in *P. aeruginosa* and 5.91% in *S. aureus*). Taken together, these data suggest that Nano-MgB_2_ induces cell membrane damage by altering the membrane potential and permeability.

To further investigate the molecular mechanism by which Nano-MgB_2_ induces bacteria death, RNA-seq was performed to screen for changes in gene expression in Nano-MgB_2_-treated *P. aeruginosa*. As shown in Supplementary Fig. 18, Nano-MgB_2_ down-regulated 1984 genes and upregulated 1918 genes. Furthermore, Kyoto Encyclopedia of Genes and Genomes (KEGG) pathway enrichment analysis of known Nano-MgB_2_-related genes was performed. Numerous important pathways such as ‘ribosome’, ‘aminoacyl-tRNA biosynthesis’, and ‘Protein export’ were enriched, and most genes in these pathways were upregulated in Nano-MgB_2_-treated *P. aeruginosa*, suggesting that Nano-MgB_2_ induced a stress response (Fig. 4i). Interestingly, the RNA degradation pathway was enriched, and 14 of the 16 related genes were upregulated after Nano-MgB_2_ treatment (Fig. 4j). Accordingly, we verified the expression of these 16 genes in *P. aeruginosa* treated with Nano-MgB_2_ using QPCR. As shown in Fig. 4k, l, Nano-MgB_2_ significantly promoted the expression of these 16 RNA degradation-related genes, consistent with the results of RNA-seq. These data suggest that Nano-MgB_2_ kills bacteria by promoting RNA degradation. Indeed, previous studies showed that RNA was degraded in response to changes in membrane permeability^40,41^. Thus, these data demonstrate that Nano-MgB_2_ reacts with LPS in the cell wall continuously releasing Mg^2+^ at the cell membrane, which disrupts the bacterial membrane, leading to RNA degradation and bacterial death (Fig. 4m).

### Nano-MgB_2_ suppressed dead bacteria- and LPS-induced inflammation in vitro

**Fig. 5:**
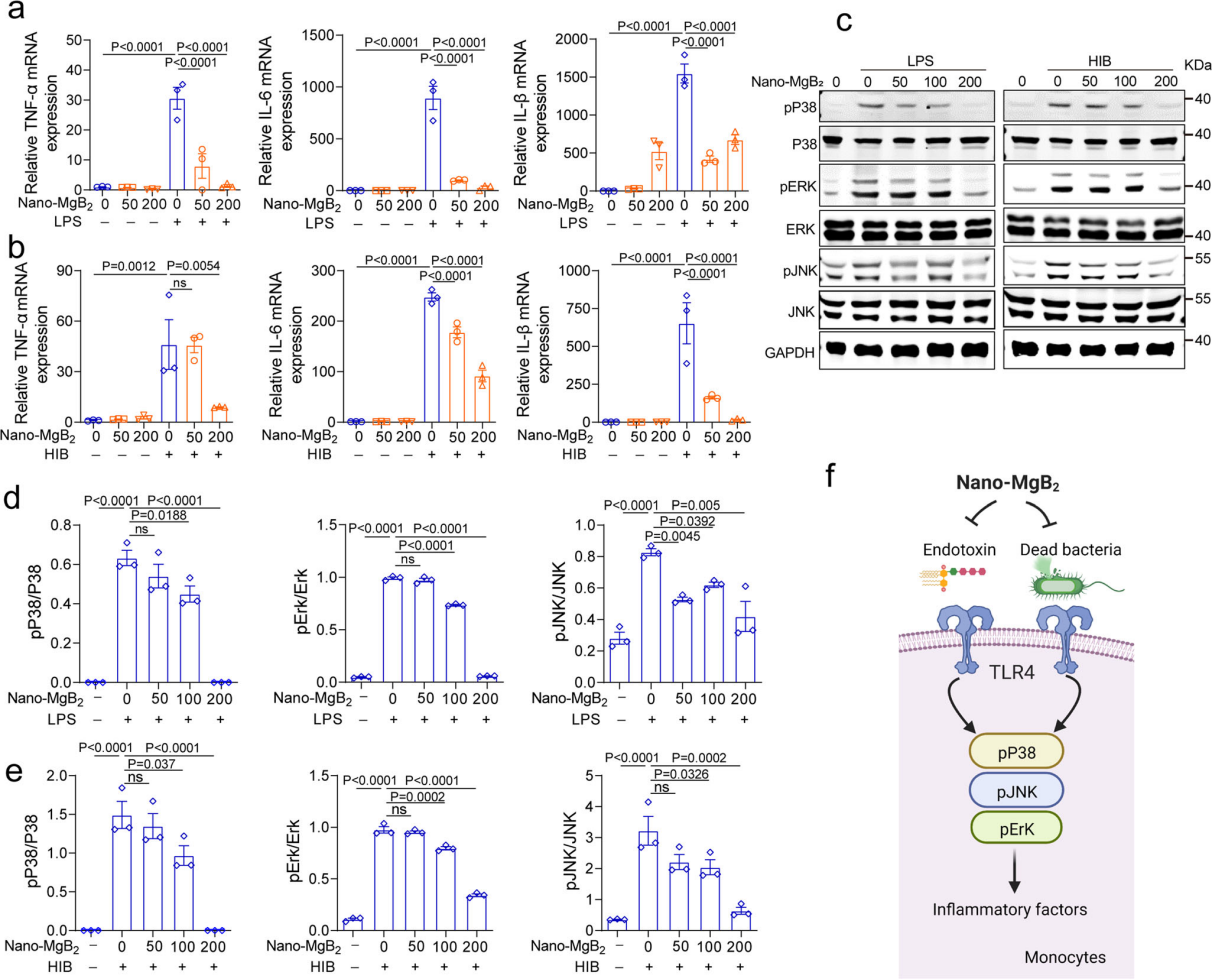
Nano-MgB_2_ inhibited LPS- and dead bacteria-induced inflammation in Macrophage. **a** QPCR of TNF-α, IL-6, and IL-1β in Raw 264.7 cells treated with 100 ng/mL LPS with 50 and 200 μg/mL Nano-MgB_2_ for 3 h (*n* = 3 biological independent cells). **b** QPCR of TNF-α, IL-6, and IL-1β in Raw 264.7 cells treated with 5 × 10^7^ dead bacteria (HIB, heat-inhibited bacteria, *P. aeruginosa*) with 50 and 200 μg/mL Nano-MgB_2_ for 3 h (*n* = 3 biological independent cells). **c** Western Blot of pP38/P38, pErk/Erk, and pJNK/JNK in Raw 264.7 cells treated as in (**a**) for 15 min. Western Blot of pP38/P38, pErk/Erk, and pJNK/JNK in Raw 264.7 cells treated as in (**b**) for 15 min. **d**, **e** Quantitative analysis of (**c**) by grayscale scanning using Image J software (*n* = 3 in each group). **f** Schematic illustration of the mechanism by which Nano-MgB_2_ inhibits dead bacteria or LPS-induced inflammation. Data are representative of at least three independent experiments with similar results. Values are the mean ± SEM. One-way ANOVA with Bonferroni post test was used to analyze multiple groups. Source data are provided as a Source data file.

To further investigate whether Nano-MgB_2_ inhibits inflammation induced by dead bacteria or dead bacteria-released endotoxin (LPS), we first assessed the toxicity of Nano-MgB_2_ towards human cell lines, including skin keratinocytes (HaCat cells) and immunocytes (Raw264.7 macrophages) using cell counting kit-8 (CCK-8). As shown in Supplementary Fig. 19, Nano-MgB_2_ was not toxic to skin keratinocytes and immunocytes after incubation for 24 h, and the components released from Nano-MgB_2_ such as Mg^2+^ and H_3_BO_3_ showed no toxicity towards keratinocytes. Next, we treated macrophage cells with LPS or dead bacteria (HIB, heat-inhibited bacteria) in the presence or absence of Nano-MgB_2_. As shown in Fig. 5a, b, LPS and HIB dramatically induced the expression of inflammation-related molecules such as tumor necrosis factor α (TNF-α), interleukin 6 (IL-6), and interleukin 1beta (IL-1β), which were significantly inhibited after treatment with Nano-MgB_2_. These results indicate that dead bacteria and dead bacteria-released endotoxin (LPS) can significantly induce a strong inflammatory response in immune cells and Nano-MgB_2_ can interact with LPS to block the inflammatory response. As previously reported, LPS can activate Toll-like receptor 4 and induce the production of inflammatory factors by regulating the mitogen-activated protein kinase (MAPK) signaling pathway^42,43^. As shown in Fig. 5c–e, LPS-induced phosphorylation of MAPK such as p38, Erk, and JNK were significantly inhibited by Nano-MgB_2_ treatment, suggesting that Nano-MgB_2_ blocks HIB/endotoxin (LPS)-induced inflammation by regulating MAPK signaling pathway (Fig. 5f).

### Nano-MgB_2_ inhibited bacterial survival in vivo

**Fig. 6:**
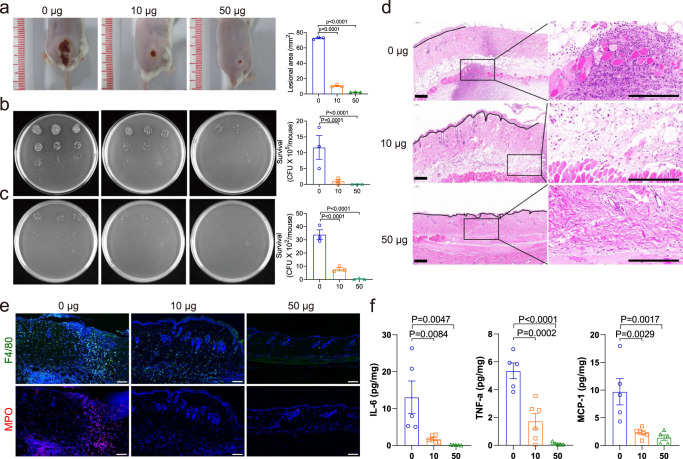
Nano-MgB_2_ inhibited skin infection in vivo. **a** Photographs of *P. aeruginosa*-infected mouse skin treated with or without 10 μg and 50 μg Nano-MgB_2_ (*n* = 3 biologically independent mice). **b**, **c** Survival of bacteria from *P. aeruginosa*-infected mouse skin or spleen taken as in (**a**) (*n* = 3 biologically independent mice). **d** H&E staining of *P. aeruginosa*-infected mouse skin taken as in (**a**). Scale bar = 200 μm. **e** Immunofluorescence staining with the neutrophil cell marker myeloperoxidase (MPO) and macrophage cell marker F4/80 of *P. aeruginosa*-infected mouse skin taken as in (**a**). Scale bar = 100 μm. **f** TNF-α, IL-6, and MCP-1 protein expression detected by CBA MOUSE INFLAMMATION kit in *P. aeruginosa*-infected mouse skin taken as in (**a**) (*n* = 5 biologically independent mice). Data are representative of at least three independent experiments with similar results. Values are the mean ± SEM. One-way ANOVA with Bonferroni post test was used to analyze multiple groups. Source data are provided as a Source data file.

To evaluate the in vivo antibacterial efficacy of Nano-MgB_2_, a *P. aeruginosa*-infected skin infection model was constructed. As shown in Fig. 6a, the *P. aeruginosa* infection-induced lesion area was significantly inhibited by Nano-MgB_2_ treatment. Consistently, the number of bacteria in the lesions was also decreased (Fig. 6b). As bacterial infections can progress from a local to systemic state, the number of bacteria in the spleen was also decreased by Nano-MgB_2_ treatment (Fig. 6c). To further investigate the effect of Nano-MgB_2_ on *P. aeruginosa*-infected skin tissue, the skin lesion areas were dissected using hematoxylin and eosin (H&E) staining. As shown in Fig. 6d, the bacteria-infected skin showed severely destroyed epithelia cells in the epidermis and increased lipogenesis and hematoxylin-positive cells in the dermis. After Nano-MgB_2_ treatment, the epidermal epithelialization was accelerated, and the lipogenesis and hematoxylin-positive cells were dramatically decreased in the dermis, indicating the decrease of inflammation. Furthermore, the granulation tissues were transferred into the scar tissue with more fibroblasts, demonstrating that the infected skin was repaired after Nano-MgB_2_ treatment. To determine whether the increased hematoxylin-positive cells were inflammatory cells, we performed immunofluorescence staining with the neutrophil cell marker myeloperoxidase (MPO)^44,45^ and macrophage cell marker F4/80^46,47^. As shown in Fig. 6e, Nano-MgB_2_ treatment significantly decreased the number of *P. aeruginosa*-induced neutrophils and macrophages, indicating decreased skin infection after Nano-MgB_2_ treatment. Consistently, inflammatory factors such as IL-6, TNF-α, and monocyte chemoattractant protein (MCP)-1 were inhibited after Nano-MgB_2_ treatment (Fig. 6f). Similar results were observed in mice infected with *S. aureus* in the presence of Nano-MgB_2_. Nano-MgB_2_ significantly inhibited the increase of lesional size in the *S. aureus*-infected skin (Supplementary Fig. 20a, b) and bacteria numbers in the skin and spleen (Supplementary Fig. 20c–f). Furthermore, inflammatory factors such as IL-6, TNF-α, and MCP-1 were significantly inhibited (Supplementary Fig. 20g, h). These data demonstrate that Nano-MgB_2_ can inhibit bacteria growth and decrease bacteria-induced inflammation in vivo.

### Nano-MgB_2_ suppressed dead bacteria-induced skin inflammation in vivo

**Fig. 7:**
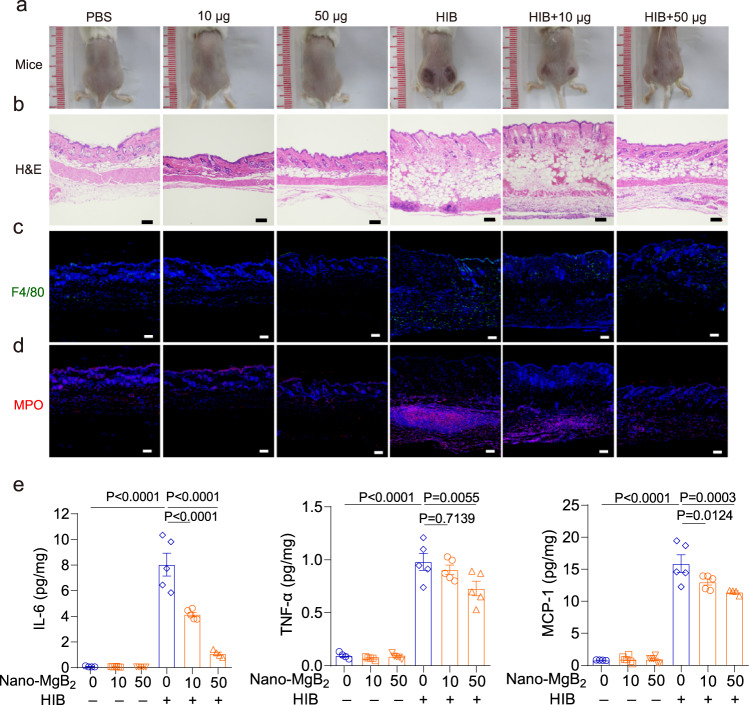
Nano-MgB_2_ inhibited dead bacteria-induced skin inflammation in vivo. **a** Photographs of dead bacteria-induced (HIB, heat-inhibited bacteria, *P. aeruginosa*) mouse skin inflammation treated with or without 10 μg and 50 μg Nano-MgB_2_ (*n* = 5). **b** H&E staining of HIB-induced mouse skin taken as in (**a**). Scale bar = 100 μm. Immunofluorescence staining with the (**c**) macrophage cell marker F4/80 and (**d**) neutrophil cell marker myeloperoxidase (MPO) of HIB-induced mouse skin taken as in (**a**). Scale bar = 100 μm. **e** IL-6, TNF-α, and MCP-1 protein expression detected by CBA mouse inflammation kit of HIB-induced mouse skin taken as in (**a**) (*n* = 5 biologically independent mice). Data are representative of at least three independent experiments. Values are the mean ± SEM. One-way ANOVA with Bonferroni post test was used to analyze multiple groups. Source data are provided as a Source data file.

Beside bacteria infection, the dead bacteria also induce excessive inflammation in the skin. For example, LPS can be released from the bacterial surface upon bacteria die or lyse. This free LPS, called endotoxin, can induce excessive inflammation and toxicity to the host. As shown in Fig. 7a, dead bacteria significantly cause the redness and swelling of the skin, and this phenomenon was dramatically inhibited after Nano-MgB_2_ treatment. To further investigate the inflammatory conditions and histopathological within the skin, the skin lesional areas were dissected for H&E and immunofluorescence staining. As shown in the H&E staining images, no obvious pathological changes in the epidermis were observed in the HIB-treated skin. Comparatively the lipogenesis and hematoxylin-positive cells were dramatically increased in the dermis, and these phenomena were dose-dependently inhibited by the Nano-MgB_2_, indicating that Nano-MgB_2_ may inhibit dead bacteria-induced inflammatory response (Fig. 7b). Consistent result was showed in immunofluorescence staining that Nano-MgB_2_ significantly decreased the number of neutrophils (MPO-positive cells) and macrophages (F4/80-positive cells) induced by dead bacteria (Fig. 7c, d). Furthermore, the inflammatory factors such as IL-6, TNF-α, and MCP-1 were also significantly inhibited after Nano-MgB_2_ treatment (Fig. 7e). All these data demonstrate that even dead bacteria can induce serious skin inflammation, and Nano-MgB_2_ can inhibit dead bacteria-induced inflammation.

### Nano-MgB_2_ promoted infected-wound healing in vivo

**Fig. 8:**
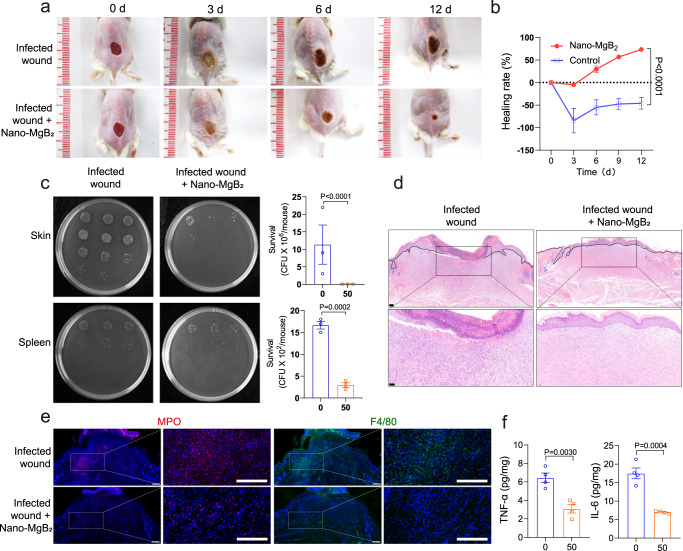
Nano-MgB_2_ enhanced infected-wound healing. **a** Photographs of *P. aeruginosa*-infected mouse skin wounds treated with or without 50 μg Nano-MgB_2_ for different days. **b** The wound healing rate of mice treated as in (**a**) (*n* = 5 biologically independent mice). **c** Survival of bacteria from *P. aeruginosa*-infected mouse skin wound or spleen taken as in (**a**) (*n* = 3 biologically independent mice). **d** H&E staining of infected wounds treated with or without Nano-MgB_2_ at day 12. Scale bar (up) = 100 μm, scale bar (down) = 50 μm. **e** Immunofluorescence staining with the neutrophil cell marker myeloperoxidase (MPO) and macrophage cell marker F4/80 of *P. aeruginosa*-infected mouse skin wounds taken as in (**a**) at day 12 (scale bar = 100 μm). **f** TNF-α and IL-6 protein expression detected by CBA mouse inflammation kit in *P. aeruginosa*-infected mouse skin wounds treated with 50 μg Nano-MgB_2_ (*n* = 4 biologically independent mice). Data are representative of at least three independent experiments with similar results. Values are the mean ± SEM. Two-way ANOVA with Bonferroni post test was used in (**b**), and t-test was used in (**c**) and (**f**). Source data are provided as a Source data file.

Because of the excellent antibacterial and anti-inflammatory activities of Nano-MgB_2_ both in vitro and in vivo, we next investigated the function of Nano-MgB_2_ in *P. aeruginosa*-infected skin wound healing. As shown in Fig. 8a, b, 50 µg of Nano-MgB_2_ clearly accelerated the healing rate of *P. aeruginosa*-infected wound. Furthermore, the number of *P. aeruginosa* in the skin and spleen was significantly reduced after Nano-MgB_2_ treatment (Fig. 8c). To further investigate the histopathological alterations within the wound, the wounds were collected on day 12 and the section was cut for H&E staining. As shown in Fig. 8d, the epidermal layer in the infected wound is still incomplete. Necrotic cells along with a large number of hematoxylin-positive cells were seen in the wound area in the infected wound. However, in the Nano-MgB_2_ treatment group, the epithelization was complete, and the wound was covered by a full layer of epidermal. Furthermore, the number of necrotic cells and the hematoxylin-positive cells was dramatically decreased, when compared with that of infected wounds. In addition, the results of immunofluorescence staining showed that Nano-MgB_2_ treatment significantly reduced the number of neutrophils (MPO-positive cells) and macrophages (F4/80-positive cells), indicating decreased bacterial infection and inflammation after Nano-MgB_2_ treatment (Fig. 8e). Moreover, inflammatory factors such as TNF-α and IL-6 were dramatically decreased after Nano-MgB_2_ treatment, further demonstrating decreased bacterial infection and inflammation in Nano-MgB_2_-treated wounds (Fig. 8f). These results are consistent with those observed in *S. aureus*-infected skin wounds treated with Nano-MgB_2_. Nano-MgB_2_ significantly promoted *S. aureus*-infected skin wound healing (Supplementary Fig. 21a, b) and inhibited bacterial growth in the skin (Supplementary Fig. 21c, d). Furthermore, hematoxylin-positive cells and inflammatory factors such as TNF-α, IL-6, and MCP-1 were significantly inhibited (Supplementary Fig. 21e, f). In addition, the mRNA expression level of the oxidative stress gene *Hmox1* was dramatically decreased in Nano-MgB_2_-treated wounds, when compared with the untreated group, suggesting that Nano-MgB_2_ decreases oxidative stress through its ROS-scavenging activity (Supplementary Fig. 22). These data demonstrate that Nano-MgB_2_ promotes wound healing by inhibiting bacterial growth, bacteria-induced excessive inflammation, and oxidative stress.

## Discussion

Bacteria and bacteria-induced excessive inflammation are involved in the healing of infected wounds. Wound treatment strategies that simultaneously inhibit both bacterial infection and dead-bacteria-induced excessive inflammation are urgently needed in the clinic. LPS/PGN is the key structural and functional component of bacteria that is crucial for ensuring bacterial survival and pathogenicity. Therefore, targeting LPS/PGN is a promising strategy for simultaneously inhibiting live bacterial infection and dead bacteria-induced excessive inflammation, leading to bacteria-infected wound healing. In this study, we proposed a boron-trapping strategy to trap LPS/PGN from escaping or moving by forming stable borate ester bonds between MB NPs and diol of LPS/PGN, resulting in excellent antibacterial and anti-inflammatory effects, finally promoting infected wound healing.

Currently, LPS-binding peptides have been reported to react with LPS or LPS-related receptors, exhibiting antibacterial or anti-inflammatory effects. For example, HVF18 (HVFRLKKWIQKVIDQFGE), a Thrombin-derived C-terminal peptide, can bind to LPS. The binding of the HVF18 to LPS competitively blocks CD14–LPS interaction, thus interfering with TLR4 dimerization and the downstream inflammatory responses^10^. However, this peptide has no antibacterial activity. In addition, antimicrobial peptides, such as cathelicidins, are able to bind to LPS, suppressing LPS-induced pro-inflammatory responses^17^. However, the proteolytic stability of peptides limits their application. Therefore, compared with LPS-binding peptides, MB NPs can consistently and stably trap LPS/PGN, exerting its excellent antibacterial and anti-inflammatory effects.

Recently, advances in nanotechnology have led to the development of metal nanomaterials and 2D materials, which exhibit antimicrobial properties. Metals such as silver and magnesium oxide have been utilized for their antimicrobial properties for thousands of years. The mechanisms responsible for the metal nanomaterials were derived from the production of ROS and the metal cations. Two-dimensional (2D) materials such as nanosheets and nanoplatelets have been reported as potential antimicrobials. The general antimicrobial mechanisms include cell membrane damage, charge transfer, ROS production, and oxidative stress. Compared with metal nanomaterials and 2D antibacterial materials, MB NPs possess the unique hydrolytic two-dimensionalization characterization and biological activity (Supplementary information Table 1). MB NPs belong to the interstitial compound, which is composed of intralayer boron-boron (B–B) covalent bonds and interlayer boron–metal (B–M) ion bonds^48^. During the hydrolysis process, the interlayer B–M bonds are preferentially broken to form boron–hydrogen (B–H) bonds, at the same time, metal ions, as well as hydroxides are released, and the morphology of MB NPs gradually becomes two-dimensional^26^. The specific surface area of MB NPs gradually increased during the hydrolysis process, which realized the continuous provision of the boron dihydroxyl groups, ensuring a long-time interaction between MB NPs and LPS/PGN. Furthermore, the gradually released hydroxides from MB NPs hydrolysis can regulate the configuration of boron atom, which is helpful to form stable borate ester bonds between MB NPs and LPS/PGN. This gradually provided boron dihydroxyl groups and the released hydroxides during the hydrolysis of MB NPs significantly improved the ability of MB NPs to trap LPS/PGN, which not only effectively enhanced the local cation concentration, destroying the bacterial cell membrane and achieving a strong antibacterial effect, but also complexed dead bacteria-released LPS and PGN to inhibit excessive inflammation, finally promoting wound healing. Taken together, the hydrolytic two-dimensional feature of MB NPs significantly enhances the biological function of MB NPs, which is distinct from other traditional metal nanomaterials and 2D antimicrobial materials, both in terms of material structure and biological mechanism.

In this study, MB NPs were synthesized using improved Self-propagating high-temperature synthesis as previously reported by our group^36^. The SHS process utilizes the exothermic heat of the reaction to synthesize materials, and its spontaneous-sustaining, fast-propagating nature endows this technique with the ability for the large-scale synthesis of MB NPs^49,50^. In addition, we can change the reaction conditions, such as gas atmosphere, or heating/cooling rate^51^, to realize the crystallization of synthetic materials in nanoscale. Therefore, these MB NPs have the prospect of clinical transformation.

In this study, MB NPs exhibited higher LPS/PGN-trapping activities than other materials containing boron dihydroxyl groups. This is because the local alkaline microenvironment generated by hydrolysis of MB NPs could enhance the stability of borate ester bonds to effectively improve the binding ability of MB NPs to bacterial polysaccharides. Boron dihydroxyl groups interact with 1,2- or 1,3-diols of polysaccharides to form cyclic esters. If the boron atom is in a planar trigonal configuration (*sp*^2^ hybridized), the corresponding boronic ester bond is affected by the relatively large ring strain, which is prone to bond breakage. If the boron atom is in a tetrahedral configuration (*sp*^3^ hybridized), the ring tension of the O–B–O bond can be effectively relieved, making the overall structure of the boronate eater bond more stable^42^. Because MB NPs can be hydrolyzed to release hydroxides, this unique feature makes the configuration of the boron atom of its hydrolyzed product change from *sp*^2^ to *sp*^3^ configuration. Furthermore, the DFT calculations show that the strength of the borate ester bonds formed by the hydrolysate of MB NPs is stronger than that of H_3_BO_3_. Therefore, MB NPs formed more stable boronic ester bonds than *sp*^2^-hybridized H_3_BO_3_.

The inhibition efficacy of Nano-MgB_2_ against Gram-positive bacteria was lower than that against Gram-negative bacteria. This is because the LPS of *P. aeruginosa* contains much more 1,2-diol or 1,3-diol compared to PGN of *S. aureus*, thus much more Nano-MgB_2_ reacted with LPS than with PGN, supporting the importance of generating borate ester bonds to achieve antibacterial and anti-inflammatory activities. In addition to the bacteria, this strategy may also be suitable for the treatment of virus infection. Trapping the key component of the virus, such as the spike glycoprotein of SARS-CoV-2, which is the shell protein of the virus and mediates the fusion of the virus membrane with the host cell membrane^17^, may provide a therapeutic method for virus infection.

Another interesting finding is that all three types of MB NPs (MgB_2_, AlB_2_, and BeB_x_) showed antibacterial activity but exhibited variable activities against *P. aeruginosa*, indicating that different ions in the NPs have different antibacterial roles. It is generally accepted that metal cations often disrupt the bacterial membrane. Consistently, our study confirmed that Nano-MgB_2_ disrupted bacterial membranes through altering the membrane potential and permeability. However, the cause of the varied antibacterial activity among these metal cations requires further investigation.

In conclusion, we introduced a boron-trapping strategy for inhibiting the survival and pathogenicity of a pathogen by trapping the key component of the pathogen. Specifically, we designed and synthesized a series of MB NPs to trap LPS/PGN, the key component of Gram-negative bacteria and Gram-positive bacteria, respectively. Both in vitro and in vivo data demonstrated that the formation of a stable borate ester bond between MB NPs and LPS/PGN not only inhibited bacterial survival but also decreased the excessive inflammation induced by dead bacteria. These effects ultimately promoted the healing of infected wounds. Our findings provide a strategy of trapping the key component of a pathogen to promote the pathogen-infected wound healing.

## Methods

### Chemical reagents

All the chemical reagents were of analytical grade and used directly without further purification. Magnesium powder (Mg, 99.9%, AR), Aluminum (Al, 99.9%, AR), Beryllium (Be, 99.9%, AR), and Boron (B, 99.9%, AR) powder were purchased from Aladdin. Anhydrous ethanol was purchased from Sigma-Aldrich. The ultrapure water used throughout the experiment was prepared by the ELGA PURELAB classic water purification system.

### Material characterization

Transmission electron microscope (TEM) images and corresponding element mapping were obtained with Thermo Fisher Scientific Tecnai G2 F30. Scanning electron microscope (SEM) images were acquired by HITACHI S-480. X-ray diffraction (XRD) data were acquired from a Rigaku D/MAX 2250 V diffractometer with a scanning rate of 5° min^−1^ in the 2*θ* range of 10–80°. Atomic force microscopy (AFM) images were obtained on a Dimension Fast Scan (Bruker) under ScanAsyst mode. The hydrodynamic size distribution was measured via dynamic light scattering (DLS) in Microtrac Nanotrac wave II. Fourier transform infrared spectroscopy (FTIR) spectra were obtained with a BRUKER TENSOR II using KBr pellets. The variation in pH of the hydrolysate of Nano-MgB_2_ nanoparticles (MB NPs) was acquired by using a pH meter (FE28, METTLER TOLEDO). Element concentration was obtained with inductively coupled plasma optical emission spectrometry (ICP-OES) by Agilent Technologies. The X-ray photoelectron spectroscopy (XPS) plots were conducted with a Thermo Fisher Scientific Escalab 250Xi, and the accurate binding energies were determined by taking the position of C 1s peak at 284.8 eV as the calibration reference. Electron spin resonance (ESR) spectra were acquired from the JEOL FA200 electron paramagnetic resonance spectrometer. The ultraviolet-visible absorption spectra (UV-vis) were recorded on the Shimadzu UV-3600 Plus spectrophotometer.

### Synthesis of MB NPs

The MB NPs (Nano-MgB_2_, Nano-AlB_2_, and Nano-BeB_x_) were synthesized by a self-propagating high-temperature synthesis (SHS) approach. Typically, 30 mM of metal powder (Mg, Al, or Be) and 40 mM of boron powder were fully ground and placed in a 15 mL corundum crucible. The mixture reacted at 800 °C under an Ar (5% O_2_) atmosphere for 3 h with a heating rate of 10 °C/min. Then the resultant products were dispersed in 200 mL anhydrous ethanol and centrifuged at 2775 × *g* for 5 min to remove large particles. Subsequently, the NPs were collected by centrifugation at 18,759 × *g* for 15 min and then washed several times with ethanol.

### The temporal variation in the pH and of Nano-MgB_2_ hydrolysate

1 mL Nano-MgB_2_ solution (1 M Nano-MgB_2_) was sealed in a dialytic bag (cutoff molecular weight: 5000 Dalton). Then the dialytic bag was placed in a beaker containing 100 mL citric acid-sodium citrate buffer solutions of various pH values (7.5, 6.5, and 4.5) and processed in a shaker at 37 °C for 24 h (shaking speed: 100 rpm). At regular intervals, the pH value was detected by a pH meter. 1 mL solution was collected to determine the release concentration of Mg and B elements by using ICP-OES, and 1 mL fresh buffer solution was returned.

### Theoretical simulation

The DFT calculations were conducted based on the Vienna Ab-inito Simulation Package (VASP). The projector-augmented wave (PAW) method and the Perdew-Burke-Ernzerhof (PBE) exchange-correlation functional supplied with the VASP package were employed for B (*s*2*p*1 06Sep2000), O (*s*2*p*4 08Apr2002), H (ultrasoft test 15Jun2001), and C (*s*2*p*2 08Apr2002). For structural optimization including Boron ring, the positions of Boron were fixed when the others were fully relaxed. The kinetic energy cutoff of the plane wave basis set was chosen to be 520 eV and the Brillouin zone integration is performed with only Gamma point due to large enough cell parameters. An energy tolerance of 1.0 × 10^−5 ^eV and a maximum displacement of 1.0 × 10^−2 ^Å were considered. The COHP was calculated using a lobster package.

### Verification of scavenging effect of Nano-MgB_2_ on hydroxyl radical (•OH)

Using 5,5-Dimethyl-1-pyrroline N-oxide (DMPO) as the spin trap, electron spin resonance (ESR) spectroscopy was used to confirm the •OH scavenging effect of Nano-MgB_2_. 100 μL citric acid-sodium citrate buffer solution was prepared (pH = 6.5, 20 μM FeSO_4_), and Nano-MgB_2_ NPs were added to the experimental group (100 μg/mL, without Nano-MgB_2_ in the control group), then 20 μL of the mixed solution was added (5 mM H_2_O_2_ and 100 mM DMPO) after 15 min. The obtained mixture solution was detected by an ESR spectrometer at room temperature.

### Verification of scavenging effect of Nano-MgB_2_ on ROS and RNS

The ability of Nano-MgB_2_ to scavenge ROS and RNS was demonstrated by PTIO• and DPPH• scavenging experiments, respectively. PTIO• radicals were dissolved with pH = 7.5 phosphate buffer at a concentration of 25 μg/mL (DPPH• radicals were dissolved in anhydrous ethanol at a concentration of 20 μg/mL), then various concentrations of Nano-MgB_2_ (from 0 to 400 μg/mL) were added. The total volume of the reaction mixture remains the same by the addition of the corresponding reagent. The mixed solution was incubated at 37 °C for 2 h in a water bath under dark conditions, the absorbance was measured at 557 nm (519 nm for DPPH•) by UV-vis absorption spectra.

### Bacterial culture and antibacterial activity test

*Staphylococcus aureus* (ST398) and *Pseudomonas aeruginosa* (PA-14) were from Jiang’s lab at East China Normal University. *S. aureus* were cultured in Trypticase Soy Broth (TSB) and *P. aeruginosa* were grown in Luria-Broth medium (LB). One day before the experiment, bacteria were incubated overnight in a culture medium at 37 °C (220 rpm shaking), and a small aliquot of cells (4%) was re-inoculated into a fresh culture medium and then grew to logarithmic phase *S. aureus*, OD = 0.6–0.8, about 10^8^ bacteria; *P. aeruginosa*, OD = 0.5, about 10^9^ bacteria. The *P. aeruginosa* were diluted to 10^8^/mL colony-forming units, and the *S. aureus* were diluted to 10^7^/mL colony-forming units. Ten microliters of live bacteria were incubated with different concentrations of Nano-MgB_2_ at 37 °C for 6 h, The bacteria from the mixture were diluted and plated on TSB or LB agar for 24 h at 37 °C, respectively. Finally, the number of colonies were counted. For antibacterial activities of the antibiotics, ten microliters of live bacteria were incubated with Nano-MgB_2_ (12.5 μg/mL for *P. aeruginosa*, 1 mg/mL for *S. aureus*) and different kinds of antibiotics (Thermo Fisher Scientific Cat GN4F, 16 μg/mL) for 6 h and 24 h. Antibiotics for *P. aeruginosa* infection include aminoglycosides (gentamicin, amikacin), carbapenems (imipenem, meropenem), cephalosporins (ceftazidime, cefepime), fluoroquinolones (ciprofloxacin, levofloxacin), penicillin with β-lactamase inhibitors (BLI) (ampicillin/sulbactam), monobactams (aztreonam); Antibiotics for *S. aureus* include cephalexin (Sangon Biotech (A600280-0005), erythromycin (Sangon Biotech A600192-0025), and mupirocin (Sangon Biotech A606674-0500).

### Live/dead fluorescent staining and Flow cytometry

*P. aeruginosa* (10^8^/mL) treated with 272 μM Nano-MgB_2_ (12.5 μg/mL) or 544 μM H_3_BO_3_ + Mg^2+^ for 3 h were stained with SYTO9 (Thermo Fisher Scientific Cat 2266591 6 μM) and Propidium iodide (Beyotime Cat ST511, 15 μM) for 15 min at room temperature in the dark. For *S. aureus*, *S. aureus* (1 × 10^8^) were incubated with 10.88 mM Nano-MgB_2_ (500 μg/mL) or 21.76 mM H_3_BO_3_ + Mg^2+^ for 3 h. The live and dead cells were visualized with confocal laser microscopy (Nikon A1 + R-980). For the Flow cytometry assay, bacteria treated with Nano-MgB_2_ and H_3_BO_3_ + Mg^2+^ for 3 h were added with 15 μM Propidium iodide and incubated for 30 min in the dark. Flow cytometry was performed using FACScan (BD LSRFortessa X-20). The gating strategy for the Flow cytometry was listd as follows. First, using Forward Scatter Area (FSA) and Side Scatter Area (SSA) to find the main group of bacteria; second, using SSA and SSH(Side Scatter High) to remove adherent bacterial populations; last, using SSA and PE to display the positive cell population of dead bacteria (Supplementary Fig. 23).

### Preparation of bacterial samples for SEM/TEM

Bacteria (1 × 10^9^) were collected and treated with 272 μM Nano-MgB_2_ (12.5 μg/mL) and 544 μM H_3_BO_3_ + Mg^2+^ for 3 h, and then washed with PBS and fixed in 2.5% glutaraldehyde solution. After being washed with PBS and dehydrated by ethanol, bacteria were analyzed with SEM (ZEISS) Gemini300 and elemental mapping (OXFORD Xplore). For TEM analysis. Bacteria were fixed with 2.5% glutaraldehyde at 4 °C and post-fixed with 1% OsO_4_. The materials were then washed three times with PBS (15 min each) and dehydrated in a graded series of ethanol (15 min for each concentration). After penetration with 100% acetone, the materials were embedded with Epon 812 and successively polymerized at 37 °C for 18 h, 48 °C for 24 h, and at 60 °C for 48 h. The embedded samples were finally ultrathin-sectioned for 70 nm and stained with uranyl acetate and lead citrate for transmission electron microscopic (JEM2100, JEOL, Japan) observation and photography. For the C and B elemental mapping, 1 × 10^9 ^*P. aeruginosa* were incubated with 272 μM Nano-MgB_2_ (12.5 μg/mL) or 544 μM H_3_BO_3_ + Mg^2+^ for 3 h; and 1 × 10^8 ^*S. aureus* were incubated with 10.88 mM Nano-MgB_2_ (500 μg/mL) or 21.76 mM H_3_BO_3_ + Mg^2+^ for 3 h, respectively. Took 2 mL samples directly to the conductive glue and sprayed gold for 45 sec using Oxford-Quorum-SC7620. Photograph the morphology and elemental of the sample using a scanning electron microscope(GeminiSEM-300/TESCAN-MIRA-LMS).

### Cytoplasmic membrane depolarization assay

The ability of Nano-MgB_2_ to alter the cytoplasmic membrane electrical potential was determined using the membrane potential-sensitive dye DiSC3(5) (3,3’-Dipropylthiadicarbocyanine Iodide; MKBio). Bacteria grown at 37 °C in LB medium to mid-logarithmic phase (OD600 = 0.5) were harvested, washed once with PBS, and resuspended by a final concentration of 2 μM DiSC3(5). The mixture was then incubated for 30 min at room temperature to enable dye uptake and fluorescence quenching. The change in fluorescence was measured immediately after the addition of 272 μM Nano-MgB_2_ and 544 μM H_3_BO_3_ + Mg^2+^ using FACScan (Beckman Coulter) for detection. The gating strategy for Flow cytometry was the same as in Live/dead fluorescent staining.

### RNA isolation and QPCR

#### RNA isolation procedure for cells

Raw264.7 cells in a 24-well plate were washed with PBS and lysed with TRIzol (Life Technologies, Cat15596026). The total RNA was separated with chloroform, precipitated by isopropanol and washed with 75% ethanol in DEPC-treated H_2_O. For tissue, the only difference from the procedure in cells is the first step. The tissue was cut into 2 mm pieces and pulverized in 1 mL TRIzol with a homogenizer at 4 °C for a total of 2 × 60 s. For bacteria, 1 mL of bacteria were collected and lysed with 100 μL lysozyme (0.4 mg/mL) for 3–5 min. After that, an Eastep Super RNA isolation kit (Shanghai Promega, LS1040) was used for RNA isolation. DNase was used to clean the genome DNA as described by the instructions.

#### Reverse transcription of RNA and QPCR

Each sample of RNA (1–5 μg) was reversed to cDNA using Hifair® III 1st Strand cDNA Synthesis SuperMix for QPCR (YESEN, CHINA 11141ES60) as described by the instructions. Finally, the cDNA was analyzed by Quantitative PCR using Hieff® QPCR SYBR® Green Master Mix (YESEN, CHINA, 11202ES03) on ABI real-time instruments (ABI 7500) as described by the instructions. The primers were synthetized by Tsingke Cp. Ltd (China) and listed in Supplementary information Table 2.

#### RNA-seq data analysis and QPCR

Total RNA was extracted using a QIAGEN RNeasy kit (catalog no. 74104) following the manufacturer’s instructions. Ribosomal RNA removal, cDNA library construction, and paired-end sequencing with NovaSeq 6000 platform (Illumina) were completed by Nanjing Personal Biotechnology Cp. Ltd (China). Gene expression values were computed from fragments per kilo bases per million fragments (FPKM) values produced by addition of a pseudocount of 1 and log2 transformation of the results. These FPKM values were used for drawing the heatmap with the pheatmap R package. Paired differential gene expression analyses were performed with DEseq R package by addition of fold change > 2 and *p*-value <0.05. Volcano plots of these differential genes were drawing with ggplots2 R package. KEGG enrichment analyses of differential genes were performed using clusterprofiler version 3.16.1. QPCR primers for *Pseudomonas aeruginosa* were synthetized by Tsingke Cp. Ltd and listed in Supplementary information Table 3.

### Western blot

*Protein from cells*. Cells in 6-well plate were lysed with 100 μL RIPA lysis buffer on ice for 20 min (RIPA buffer: 150 mM NaCl, 1% IGEPAL CA-630, 0.5% sodium deoxycholate, 0.1% SDS, 50 mM Tris-HCl, pH 8.0 and protease inhibitors). Protein was collected from the supernatant by centrifuging at 10,625 × *g* at 4 °C for 20 min in a microcentrifuge. *Protein from tissues*. Equal amounts of lesional skins (infected skin (0.2 g), wounded skin (0.1 g)) were collected and cut into small pieces. The samples were added with ice-cold lysis buffer (RIPA, infected skin (1 mL), wounded skin (0.5 mL)) and homogenized with an electric homogenizer at 4 °C for a total of 2 × 60 s. Protein was collected from the supernatant by centrifuging at 10,625 × *g* at 4 °C for 20 min in a microcentrifuge.

The protocol for western blot was performed as follows^52^. Equal amounts of protein (20-30 μg) were reduced and denatured with 5×laemmli sample buffer, separated by SDS-PAGE gel, and transferred to the nitrocellulose membrane. After that, the membrane was blocked by 5% milk, incubated with the first antibody and second antibody as described by the manufacturer’s instructions. Finally, the membranes were developed by the Odyssey LI-COR instrument. The antibodies from Abways technology (China Inc.) were listed as follows: P38 antibody (Cat:CY5488, 1:1000); Pp38 antibody (Cat:CY6391, 1:1000; ERK1/2 antibody (Cat:CY5487, 1:1000); pErk1/2 antibody (Cat:CY5277, 1:1000); JNK1/2/3 antibody (Cat:CY5490, 1:1000); pJNK1/2/3 antibody (Cat:CY5541, 1:1000); GAPDH (Cat: AB0037, 1:5000). All antibodies were diluented by Antibody Diluent (Cat:P0023A, Beyotime (China Inc.). All the uncropped blots were supplied in the Source data file.

### H&E and IF

The samples from the skin wound were dissected and fixed in 4% paraformaldehyde. After dehydrated in gradient alcohol, the samples were embedded in paraffin. Five micrometers of tissue sections were cut and mounted on glass slides. For H&E staining, the sections were dewaxed in xylene, rehydrated in gradient alcohol, washed briefly in distilled H_2_O, stained in Harris hematoxylin solution, differentiated with 0.3% acid alcohol, and stained with eosin. After dehydrating in gradient alcohol again, the sections were cleared in xylene and mounted with a xylene-based mounting medium. For immunofluorescence assays, the sections were pretreated with antigen retrieval solution (pH = 9.0 EDTA solution) after dewaxed in xylene, stained with indicated first antibodies (MPO (Abcam ab208670, 1:100); mF4/80(Santacruz (BM8), sc-52664, 1:500) and second antibodies Alexa Fluor 594 donkey anti-rabbit, Alexa Fluor 488 donkey anti-rat according to the manufacturer’s introduction, and then mounted with DAPI-contained mounting solution. The images were scanned by 3DHISTECH CaseViewer 2.4.

### Inflammation cytokine detection

Protein was collected as mentioned above. TNF-α, IL-6, and MCP-1 concentrations were detected by BD cytometric bead array (CBA) mouse inflammation kit (Catalog No. 552364) using BD FACSCalibur. The samples were prepared as follows: added 50 µL the mixed Capture Beads and 50 μL of sample to assay tubes, then added 50 µL the Mouse Inflammation PE Detection Reagent to each tube. Incubated for 2 h at room temperature in the dark. Added 1 mL wash buffer and centrifuged at 200 × *g* for 5 min. Resuspended in 300 μL wash buffer. Detected by flow cytometry and analyzed by FCAP Array software.

### Animals

Balb/c mice (7–8 weeks) were purchased from Charles River (CHINA Inc). All the experiments were bred separately and housed in the specific pathogen-free (SPF) animal facilities in the East China Normal University (light/dark cycle 10 h:14 h, temperature 20–26 °C, humidity 40–70%). All mouse experiments performence were approved by the East China Normal University Animal Care and Use Committee, and the ethics number is m20170215. All the surgeries were performed under a general anesthetic condition to minimize suffering. All the mice used in the experimental groups were randomly assigned.

### Cutaneous bacteria and heat-inhibited bacteria (HIB) infection in mice

The dorsal of mice were shaved and hair was removed by using chemical depilation (VEET, CHINA). For bacteria infection^53^, bacteria in the logarithmic phase (*S. aureus*, OD = 0.6–0.8, about 10^8^ bacteria; *P. aeruginosa*, OD = 0.5, about 10^9^ bacteria) were collected and 10^6^ *S. aureus* or 10^7 ^*P. aeruginosa* were intradermally injected into mouse dorsal skin and then intradermally treated with indicated concentrations of Nano-MgB_2_; For heat-inhibited bacteria infection^54^, 2 × 10^9^ CFU/mL *P. aeruginosa* were washed with PBS and killed at 70 °C for 1 h in 1 mL PBS. The bacteria were diluted and plated on TSB or LB agar to make sure all the bacteria were totally killed. 2 × 10^8^ CFU heat-inhibited *P. aeruginosa* in 100 μL PBS with or without Nano-MgB_2_ were intradermally injected into mouse dorsal skin. The lesions were photographed and measured by Image J software. Mice were euthanized on day 3 and the lesional skins were collected and homogenized to determine the survival number of bacteria. In some experiments, the lesional skins were collected for H&E staining, QPCR, and inflammation cytokine detection.

### Cutaneous-infected wound in mice

Mouse skins were shaved as mentioned above. Excisional dorsal skin wounds were made with an 8 mm sterile biopsy punch as previous report and then infected with 10^6^ *S. aureus* or 10^7 ^*P. aeruginosa* in the presence or absence of Nano-MgB_2_^55^. The wounds were finally covered with a special kind of plastic sticker (Tegaderm Film, 3M, XH003801525), the wound areas were photographed at the indicated times and calculated by Image J. The mice were euthanized and the skin surrounding the wound edges was collected for H&E staining, QPCR, and Elisa. In some experiments, the whole lesional skins were collected and homogenized to determine the survival number of bacteria.

### Statistical analysis and reproducibility

The GraphPad Prism 8.0 was used for statistical analysis. All data are representative of at least three independent experiments with similar results, and the data are presented as means ± SEM. A two-tailed t-test was used to determine the significance between the two groups. One-way or two-way ANOVA with Bonferroni post test was used to analyze multiple groups. For all the statistical tests, *P* values <0.05 were considered to be statistically significant.

### Reporting summary

Further information on research design is available in the Nature Portfolio Reporting Summary linked to this article.

## Supplementary information

Supplementary Information

Reporting Summary

## Data Availability

The main data supporting the results in this study are available within the paper and its Supplementary Information. All data generated in this study are available from the corresponding authors. The RNA-seq data are deposited in the Gene Expression Omnibus (GEO) database under the identification number GSE218031. These data can be accessed by https://www.ncbi.nlm.nih.gov/geo/query/acc.cgi?acc=GSE218031. The source data of linear graph and column diagram are provided as a Source data file 1. The source data of uncropped scans of Western blots in the figures are provided as a Source data file 2. Source data are provided with this paper.

## Source data

Source Data
