# Supplementary material for: Reactive metal boride nanoparticles trap lipopolysaccharide and peptidoglycan for bacteria-infected wound healing
Source: Nat Commun. 2022 Nov 29;13:7353. doi: 10.1038/s41467-022-35050-6 (PMC9708144; doi:10.1038/s41467-022-35050-6)
Supplement: Supplementary file 1 — Supplementary Information [file 41467_2022_35050_MOESM1_ESM.pdf]

Supplementary Information

Reactive Metal Boride Nanoparticles Trap Lipopolysaccharide and Peptidoglycan for Bacteria-Infected Wound Healing

Yun Meng[1,2#], Lijie Chen [2#], Yang Chen[1,3#], Jieyun Shi[1], Zheng Zhang[4], Yiwen Wang[4], Fan Wu[2], Xingwu Jiang[2], Wei Yang[1], Li Zhang [1], Chaochao Wang[1], Xianfu Meng[1], Yelin Wu[1*], Wenbo Bu[1,2*]

[1]Tongji University Cancer Center, Shanghai Tenth Peoples Hospital, Tongji University School of Medicine, Shanghai 200072, P. R. China

[2]Department of Materials Science and State Key Laboratory of Molecular Engineering of Polymers, Fudan University, Shanghai 200433, P. R. China

[3] School of Life Sciences and Technology, Tongji University, Shanghai 200092, P. R. China

[4]School of life Science, East China Normal University, Shanghai 200241, P. R. China

[#]These authors contributed equally to this work.

Email: sk_wuyelin@tongji.edu.cn; wbbu@fudan.edu.cn;

Supplementary Information:

Supplementary Figures 1-23

Supplementary Tables 1, 2, 3

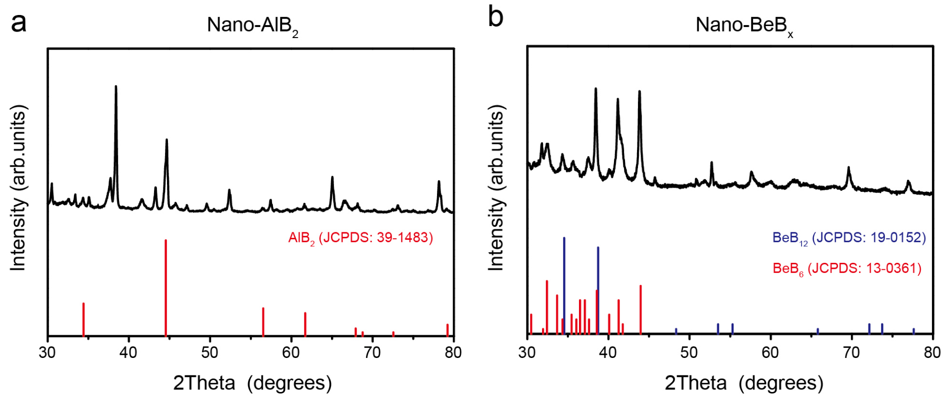

Supplementary Fig. 1 XRD patterns of **a**, Nano-AlB_2 and **b**, Nano-BeB_x. Source data are provided as a Source Data file.

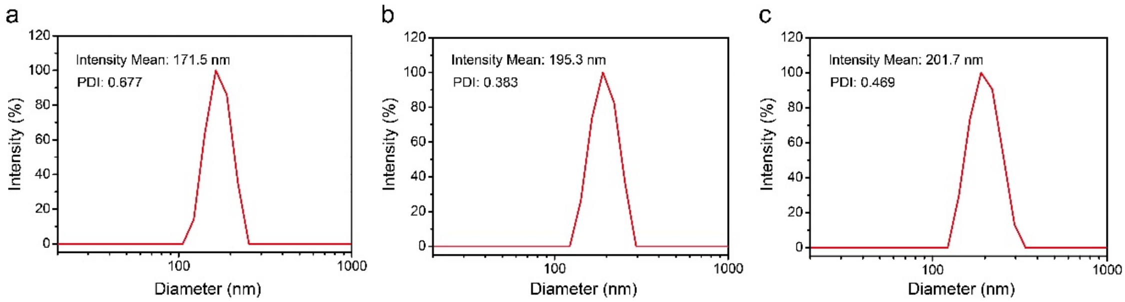

Supplementary Fig. 2 Particle size analysis of **a**, Nano-MgB_2, **b**, Nano-AlB_2 and **c**, Nano-BeB_x. Source data are provided as a Source Data file.

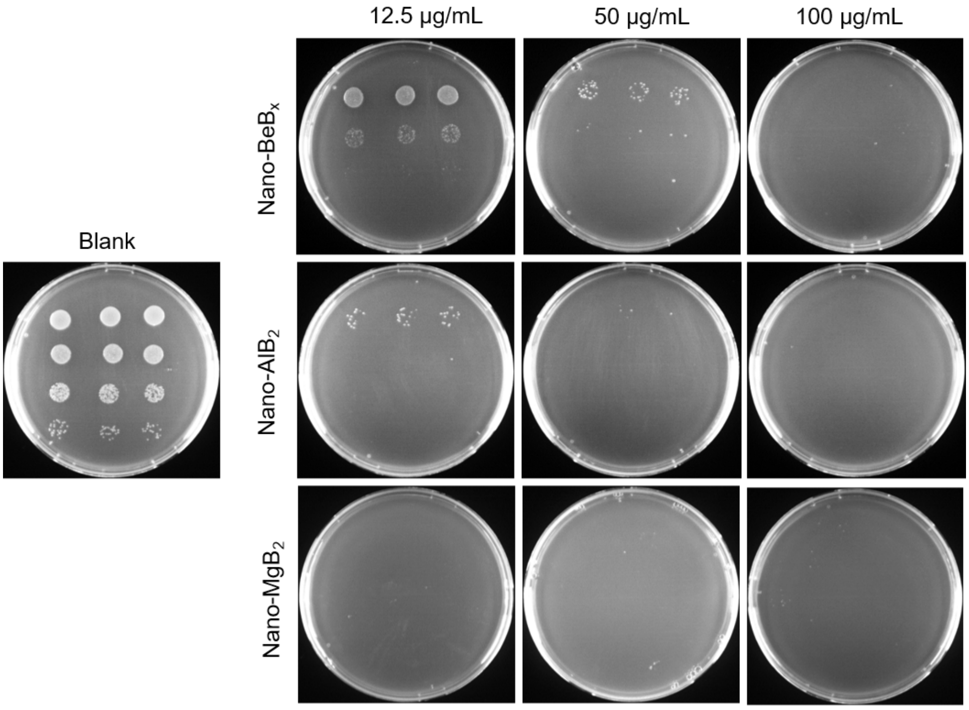

Supplementary Fig. 3 Antibacterial activity of Nano-MgB_2, Nano-AlB_2 and Nano-BeB_x. *P. aeruginosa* treated with different concentrations of Nano-MgB_2, Nano-AlB_2 and Nano-BeB_x (n=3 biological independent cells).

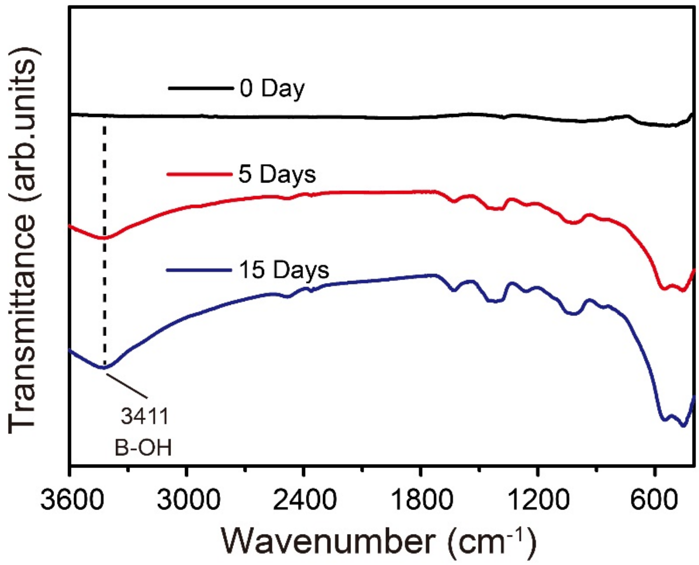

Supplementary Fig. 4 FTIR spectrum analysis of the hydrolysis process of Nano-MgB_2 in 15 days. Source data are provided as a Source Data file.

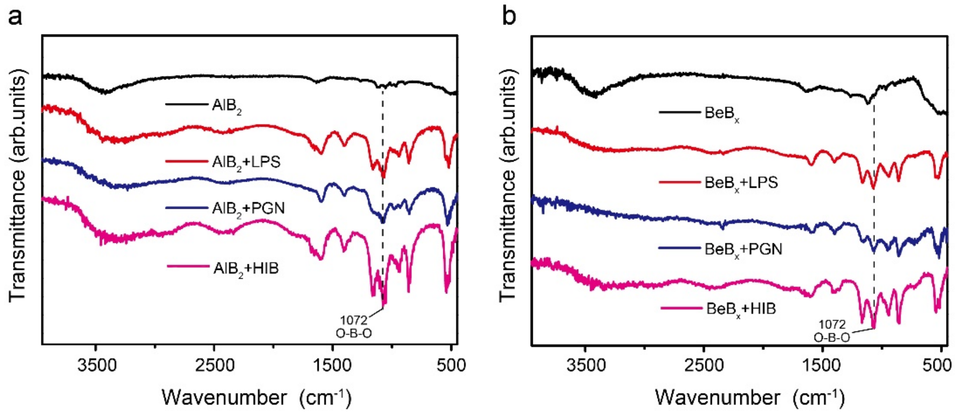

Supplementary Fig. 5 FTIR spectra of O-B-O of **a**, Nano-AlB_2 and **b**, Nano-BeB_x incubated with LPS, PGN, and HIB, respectively. Source data are provided as a Source Data file.

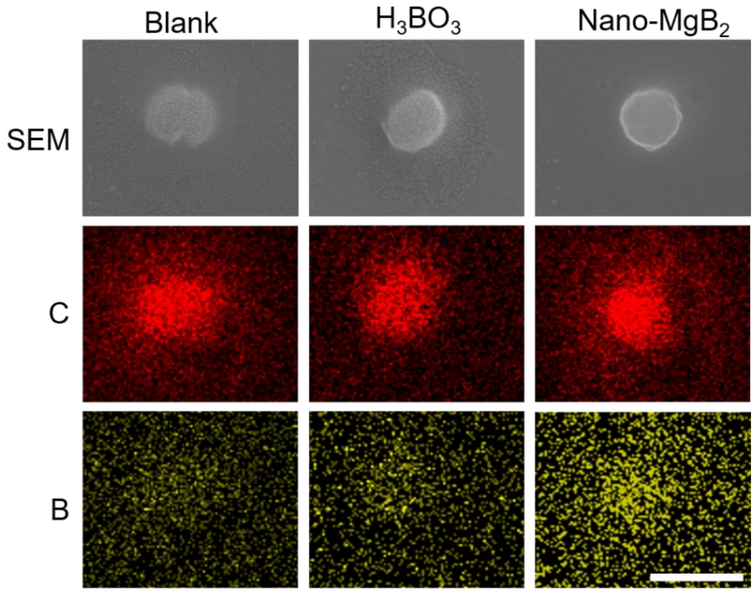

Supplementary Fig. 6 Elemental mapping of *S. aureus* incubated with H_3BO_3 and Nano-MgB_2 for 3 h (scale bar=500 nm). Data are representative of at least three independent experiments with similar results.

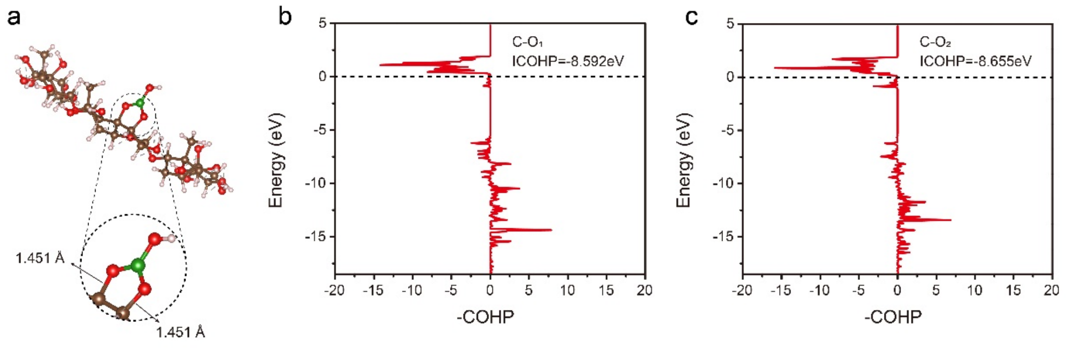

Supplementary Fig. 7 Theoretical simulation for the H_3BO_3-saccharide complexation. **a**, DFT calculations and **b-c**, COHP analysis of C-O_1, C-O_2 bond interactions corresponding to the complexation of H_3BO_3 with the 3, 4-o-hydroxyl groups of bicyclic monosaccharide structure. Source data are provided as a Source Data file.

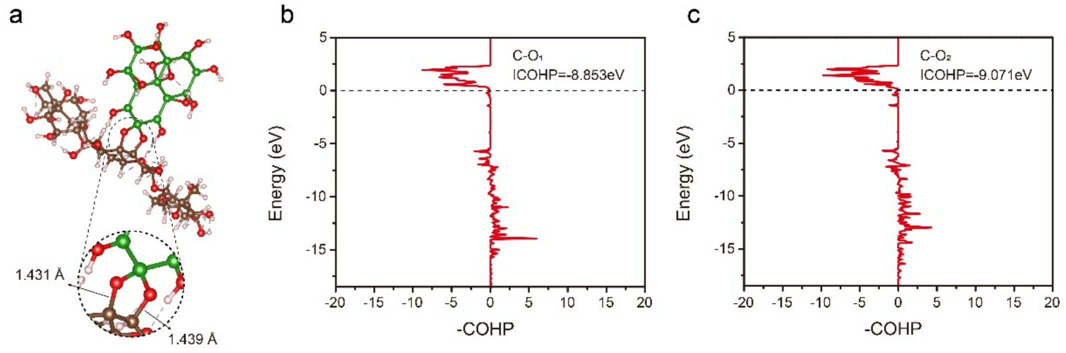

Supplementary Fig. 8 Theoretical simulation for the Nano-MgB_2-saccharide complexation. **a**, DFT calculations and **b-c**, COHP analysis of C-O_1, C-O_2 bond interactions corresponding to the complexation of hydrolysate of Nano-MgB_2 with the 3, 4-o-hydroxyl groups of bicyclic monosaccharide structure. Source data are provided as a Source Data file.

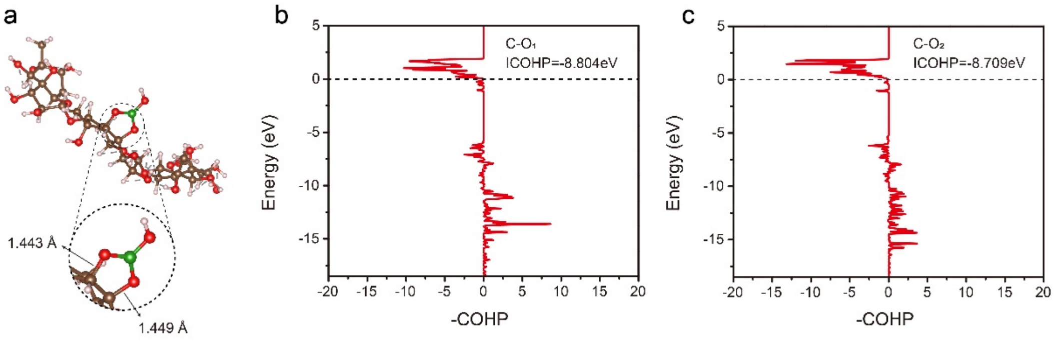

Supplementary Fig. 9 Theoretical simulation for the H_3BO_3-saccharide complexation. **a**, DFT calculations and **b-c**, COHP analysis of C-O_1, C-O_2 bond interactions corresponding to the complexation of H_3BO_3 with the 4, 5-o-hydroxyl groups of bicyclic monosaccharide structure. Source data are provided as a Source Data file.

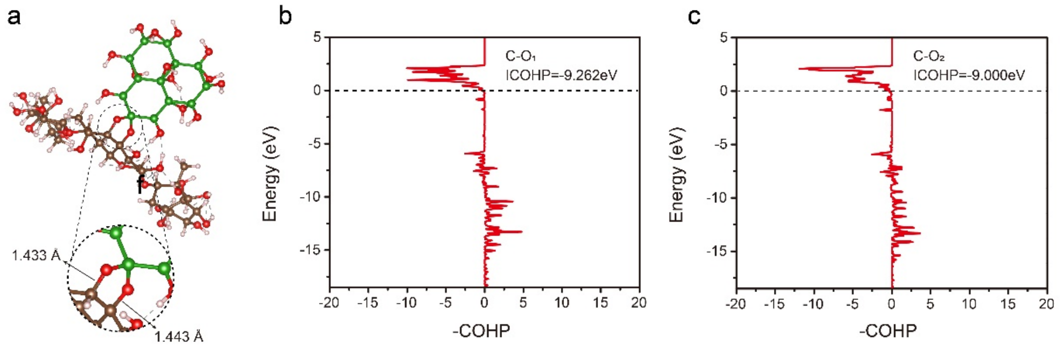

Supplementary Fig. 10 Theoretical simulation for the Nano-MgB_2-saccharide complexation. **a**, DFT calculations and **b-c**, COHP analysis of C-O_1, C-O_2 bond interactions corresponding to the complexation of hydrolysate of Nano-MgB_2 with the 3, 4-o-hydroxyl groups of bicyclic monosaccharide structure. Source data are provided as a Source Data file.

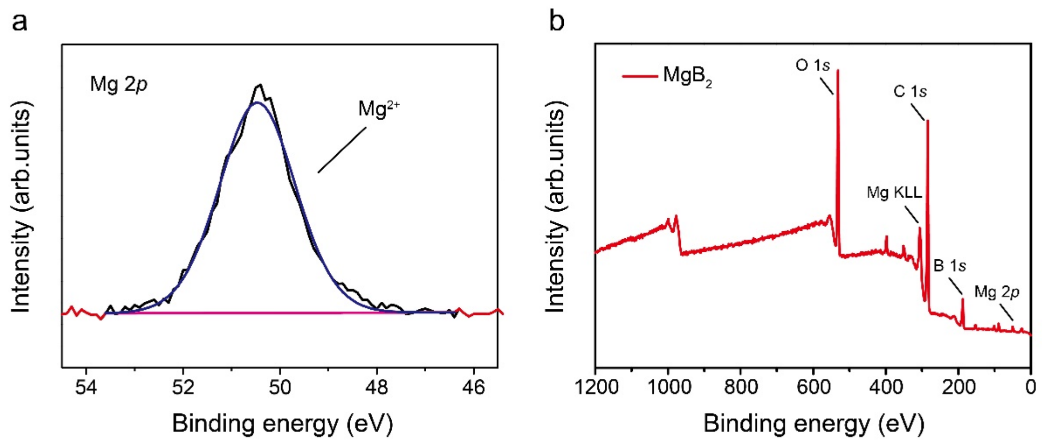

Supplementary Fig. 11 XPS characterization of Nano-MgB_2. **a**, XPS fine spectra of Mg 2*p* signal and **b**, XPS survey spectra of Nano-MgB_2 deconvoluted by the multi-Gaussian function.

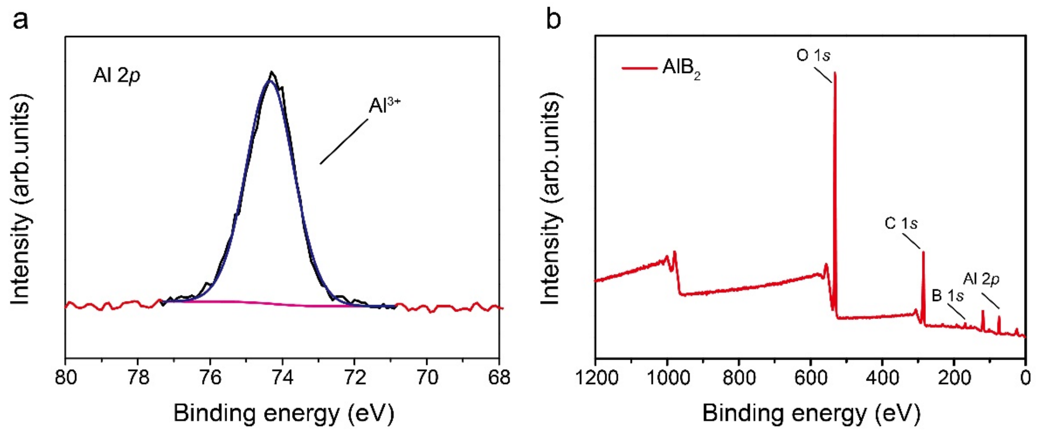

Supplementary Fig. 12 XPS characterization of Nano-AlB_2. **a**, XPS fine spectra of Al 2*p* signal and **b**, XPS survey spectra of Nano-AlB_2 deconvoluted by the multi-Gaussian function.

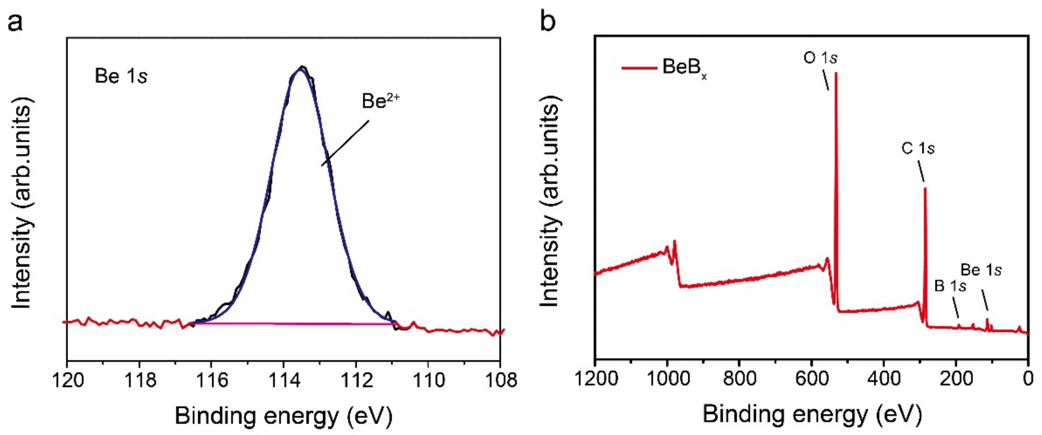

Supplementary Fig. 13 XPS characterization of Nano-BeB_x. **a**, XPS fine spectra of Be 1*s* signal

and **b**, XPS survey spectra of Nano-BeB_x deconvoluted by the multi-Gaussian function.

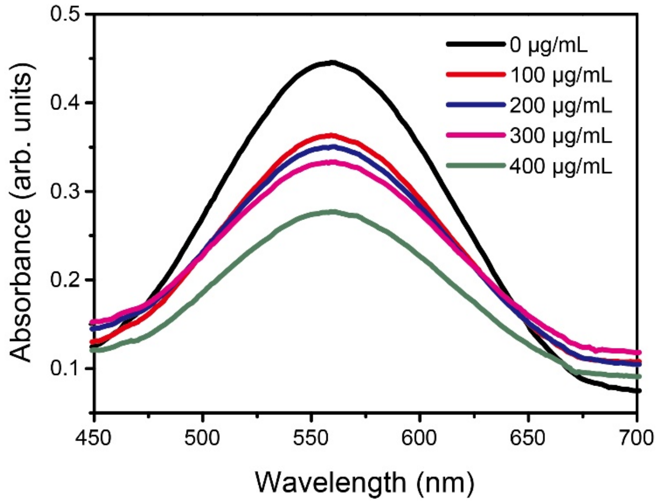

Supplementary Fig. 14 UV-vis spectra of PTIO• scavenging ability with different concentrations of Nano-MgB_2. Data are representative of at least three independent experiments. Source data are provided as a Source Data file.

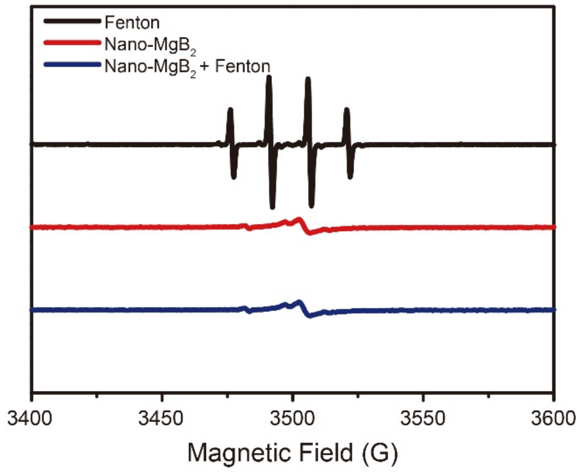

Supplementary Fig. 15 ESR spectra of Nano-MgB_2 under different conditions. Source data are provided as a Source Data file.

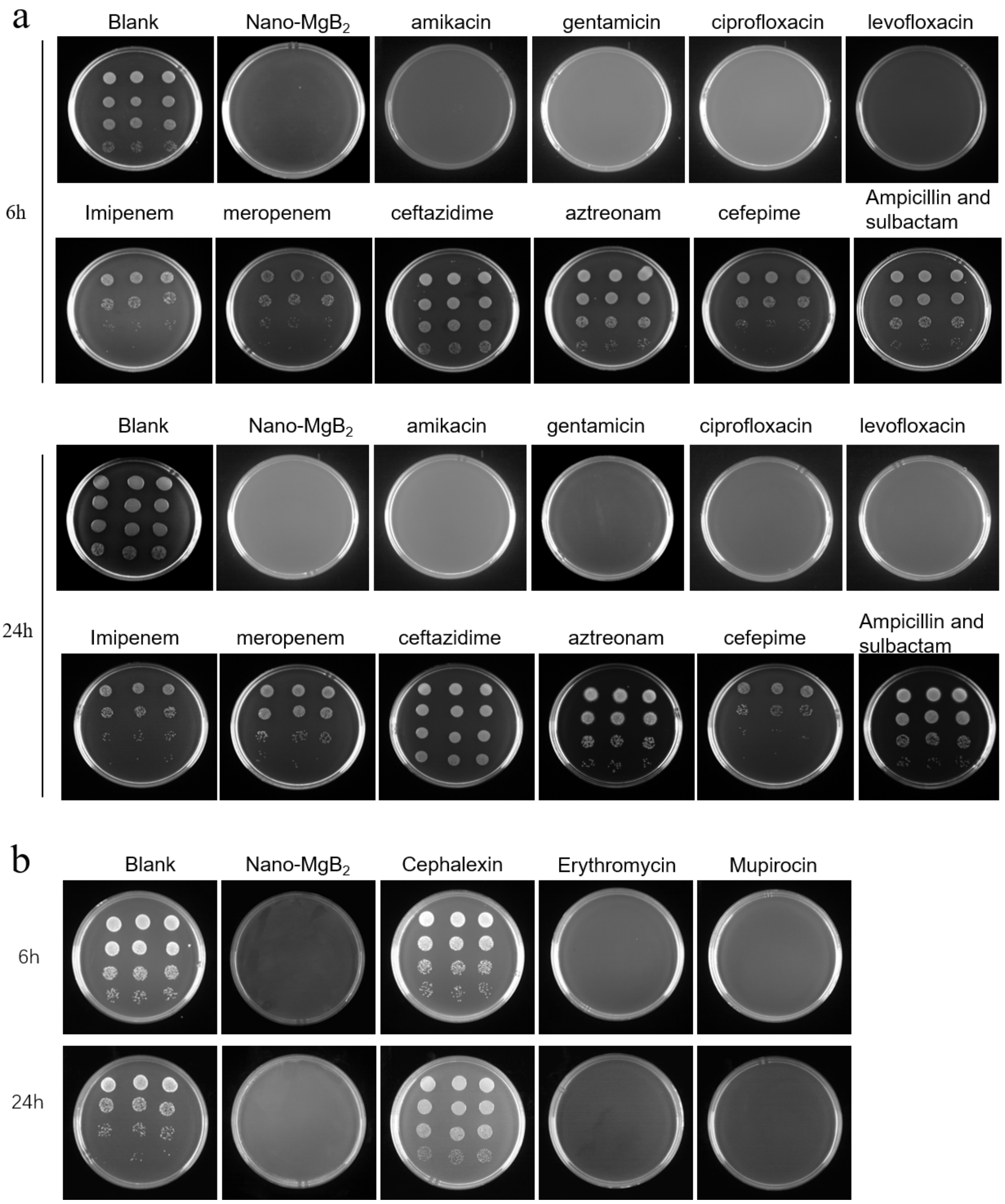

Supplementary Fig. 16 Antibacterial activity of Nano-MgB_2 and antibiotics. **a**, *P. aeruginosa* treated with Nano-MgB_2 (12.5 μg/mL) and different kinds of antibiotics (16 μg/mL) for 6 h and 24 h. **b**, *S. aureus* treated with Nano-MgB_2 (1 mg/mL) and different kinds of antibiotics (1 mg/mL) for 6 h and 24 h (n=3 biological independent cells).

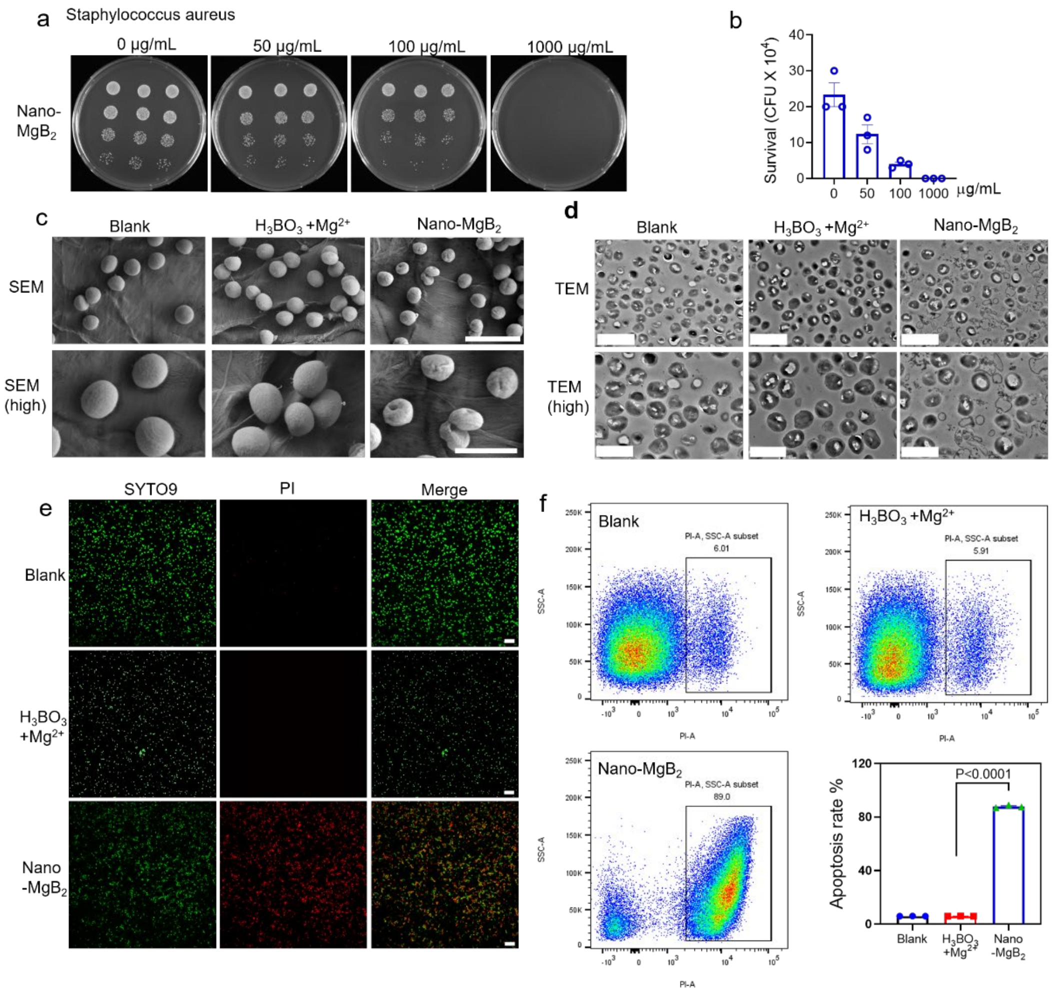

Supplementary Fig. 17 Antibacterial activity of Nano-MgB_2 against *S. aureus*. **a**, *S. aureus* treated with different concentrations of Nano-MgB_2 using colony-forming units counting method. **b**, Survival rates of *S. aureus* taken as in (**a**) (n=3 biologically independent cell). **c**, SEM images of *S. aureus* subjected to Nano-MgB_2 and H_3BO_3+Mg^{2+} Scale bar (up)=2 μm, scale bar (down)=1 μm. **d**, TEM images of *S. aureus* subjected to Nano-MgB_2 and H_3BO_3+Mg^{2+}. Scale bar (up)=2 μm, scale bar (down)=1 μm. **e**, Cell membrane permeability of *S. aureus* treated with Nano-MgB_2 (500 μg/mL) and H_3BO_3+Mg^{2+} by SYTO9 and PI staining. All cells were labelled by the membrane-permeable SYTO9 (green), whereas only cells with damaged membrane were positive for PI (red). Scale bar=20 μm. **f**, FACS analysis of PI-positive bacteria treated as in (**e**) (n=3 biologically independent cells). Data are representative of at least three independent experiments with similar results. Values are the mean ± SEM, one-way ANOVA with Bonferroni post-test was used to analyze multiple groups. Source data are provided as a Source Data file.

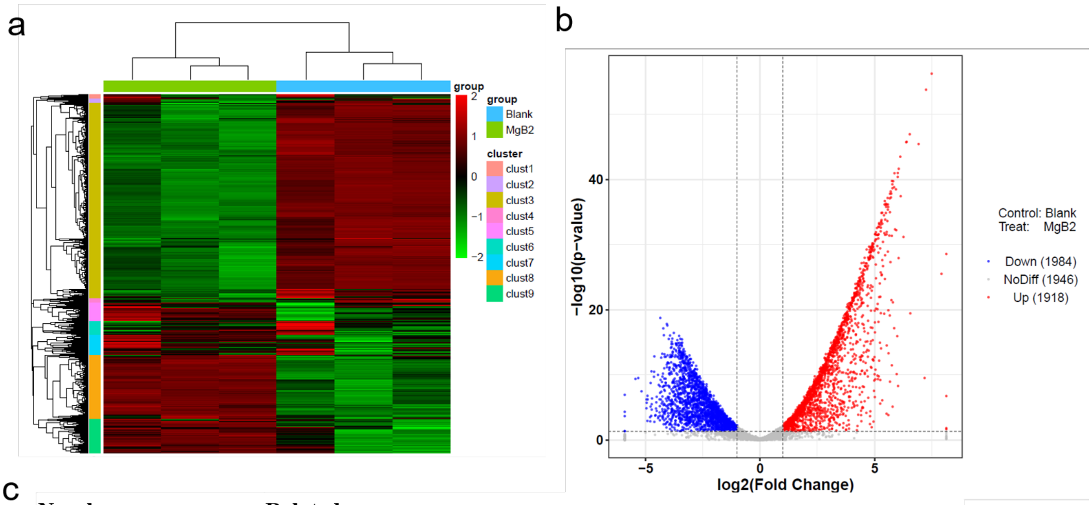

c

**Number**	**Related gene**
gene-CIA_RS23525	ATP-dependent DNA helicase RecQ [Pseudomonas aeruginosa PAO1]
gene-CIA_RS29995	Nudix hydrolase YgdP [Pseudomonas aeruginosa]
gene-CIA_RS20225	DNA helicase [Pseudomonas aeruginosa UCBPP-PA14]
gene-CIA_RS26230	hypothetical protein [Pseudomonas sp. 2_1_26]
gene-CIA_RS24915	phosphopyruvate hydratase [Pseudomonas aeruginosa PAO1]
gene-CIA_RS33410	polyphosphate kinase [Pseudomonas aeruginosa UCBPP-PA14]
gene-CIA_RS35865	molecular chaperone DnaK [Pseudomonas aeruginosa PAO1]
gene-CIA_RS36040	poly(A) polymerase [Pseudomonas aeruginosa UCBPP-PA14]
gene-CIA_RS20885	ATP-dependent RNA helicase [Pseudomonas aeruginosa PAO1]
gene-CIA_RS33425	transcription termination factor Rho [Pseudomonas aeruginosa PAO1]
gene-CIA_RS21575	ribonuclease E [Pseudomonas aeruginosa UCBPP-PA14]
gene-CIA_RS34960	exoribonuclease RNase R [Pseudomonas aeruginosa UCBPP-PA14]
gene-CIA_RS29525	ATP-dependent RNA helicase [Pseudomonas aeruginosa UCBPP-PA14]
gene-CIA_RS35975	polynucleotide phosphorylase [Pseudomonas aeruginosa PAO1]
gene-CIA_RS08900	molecular chaperone GroEL [Pseudomonas aeruginosa PAO1]
gene-CIA_RS34920	RNA-binding protein Hfq [Pseudomonas aeruginosa PAO1]

Supplementary Fig. 18. RNA seq of *P. aeruginosa* treated with Nano-MgB_2. **a**, Heat map of differential expressed genes after *P. aeruginosa* treated with Nano-MgB_2. **b**, Volcano plots comparing gene expression after *P. aeruginosa* treated with Nano-MgB_2. The P values were calculated using the negative binomial distribution. **c**, The related gene name of Fig 4j.

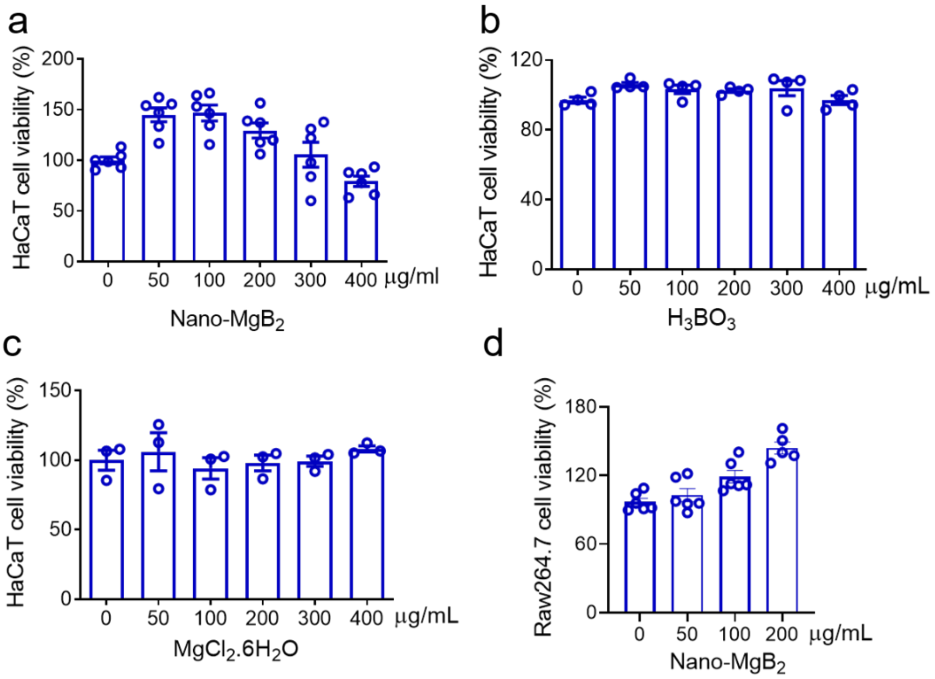

Supplementary Fig. 19 Biocompatibility of Nano-MgB_2. **a-c**, HaCaT cells treated with different concentrations of Nano-MgB_2 (n=6 biologically independent cells), H_3BO_3 (n=4 biologically independent cells) and $MgCl_2$. (n=3 biologically independent cells). **d,** Raw 264.7 cells treated with different concentrations of Nano-MgB_2 (n=5 biologically independent cells). Data are representative of at least three independent experiments with similar results. Values are the mean ± SEM. Source data are provided as a Source Data file.

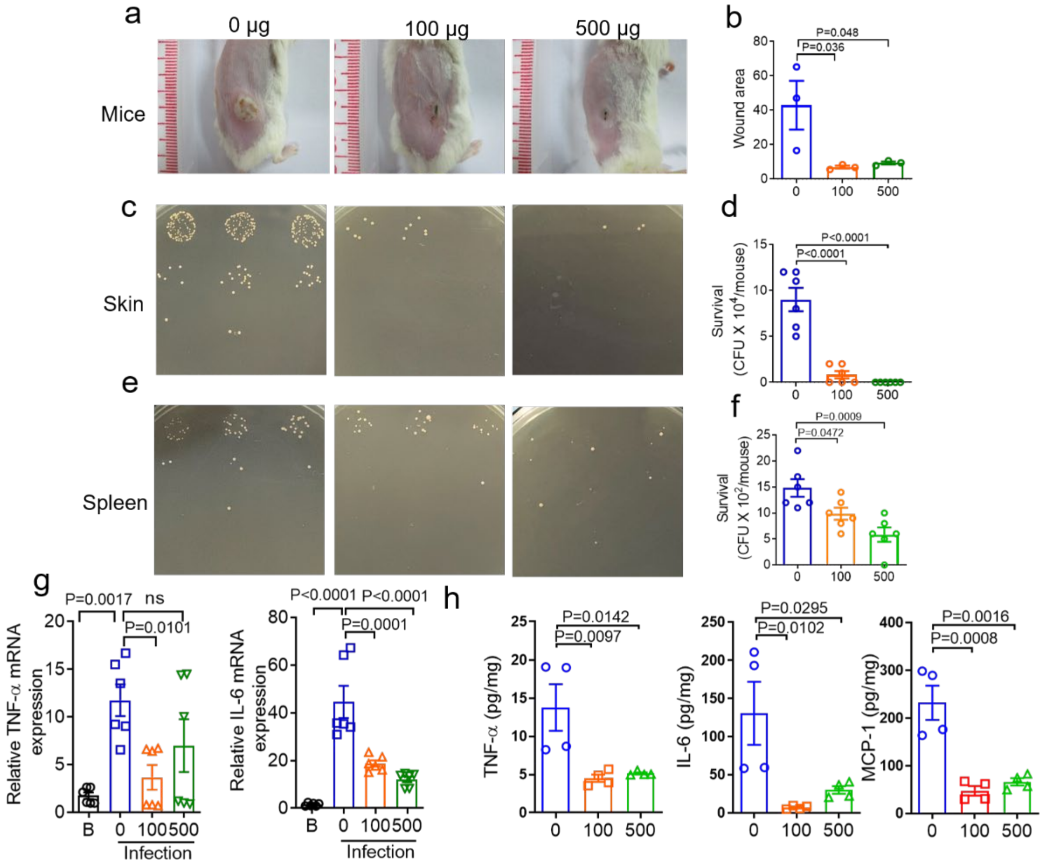

Supplementary Fig. 20 Nano-MgB_2 inhibited *S. aureus*-induced skin infection and inflammation. **a**, Photographs of *S. aureus*-infected mouse skin treated with or without 100 μg and 500 μg/mouse Nano-MgB_2 (n=3 biologically independent mice). **b**, The wound healing rate of mice treated as in (**a**) (n=3 biologically independent mice). **c**, **d**, Survival of bacteria from *S. aureus*-infected mouse skin or (**e**, **f**,) spleen taken as in (**a**) (n=6 biologically independent mice). **g**, QPCR of TNF-α and IL-6 in *S. aureus*-infected mouse skin taken as in (**a**) (n=6 biologically independent mice). **h**, TNF-α, IL-6 and MCP-1 protein expression in *S. aureus*-infected mouse skin taken as in (**a**) (n=4 biologically independent mice). Data are representative of at least three independent experiments with similar results. Values are the mean ± SEM, one-way ANOVA with Bonferroni post test was used to analyze multiple groups. Source data are provided as a Source Data file.

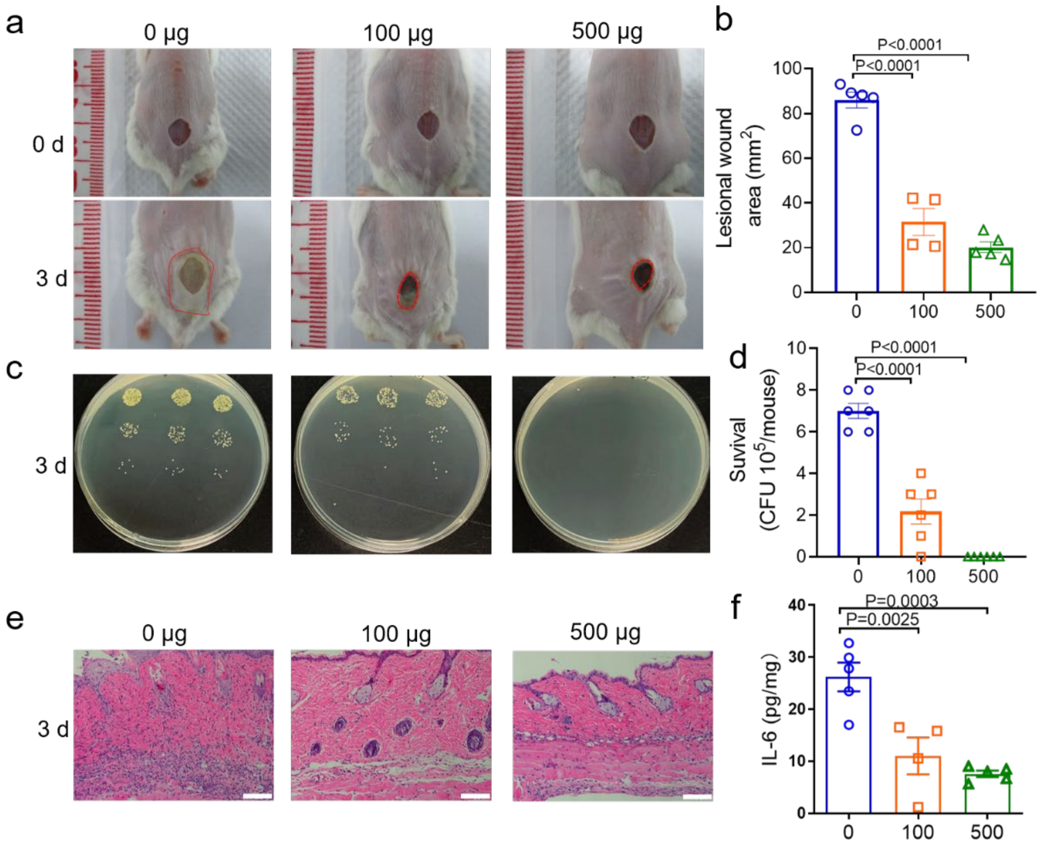

Supplementary Fig. 21. Nano-MgB_2 enhanced *S. aureus*-infected skin wound healing. **a**, Photographs of *S. aureus*-infected skin wounds topically treated with 100 μg and 500 μg/mouse Nano-MgB_2 for 3 days. **b**, The wound healing rate of mice treated as in (**a**) (n=5 biologically independent mice). **c**, **d**, The survival of *S. aureus* in *S. aureus*-infected skin wounds (n=6 biologically independent mice). e, The H&E staining of the infected wound areas (Scale bar=100 μm). **f**, The Inflammatory factor IL-6 protein expression in the lesional skins from mice treated as in (**a**) (n=5 biologically independent mice). Data are representative of at least three independent experiments with similar results. Values are the mean ± SEM, one-way ANOVA with Bonferroni post test was used to analyze multiple groups. Source data are provided as a Source Data file.

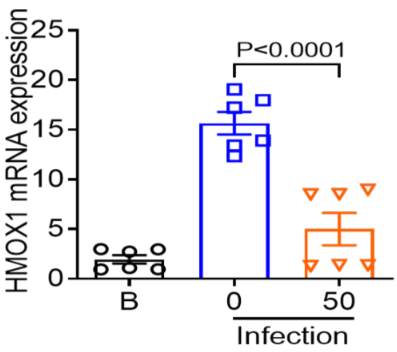

Supplementary Fig. 22. Nano-MgB_2 inhibited oxidative stress-related gene *hmox1* expression in

P. aeruginosa-infected skin wound (n=6 biologically independent mice). Data are representative of at least three independent experiments with similar results. Values are the mean ± SEM, one-way ANOVA with Bonferroni post test was used to analyze multiple groups. Source data are provided as a Source Data file.

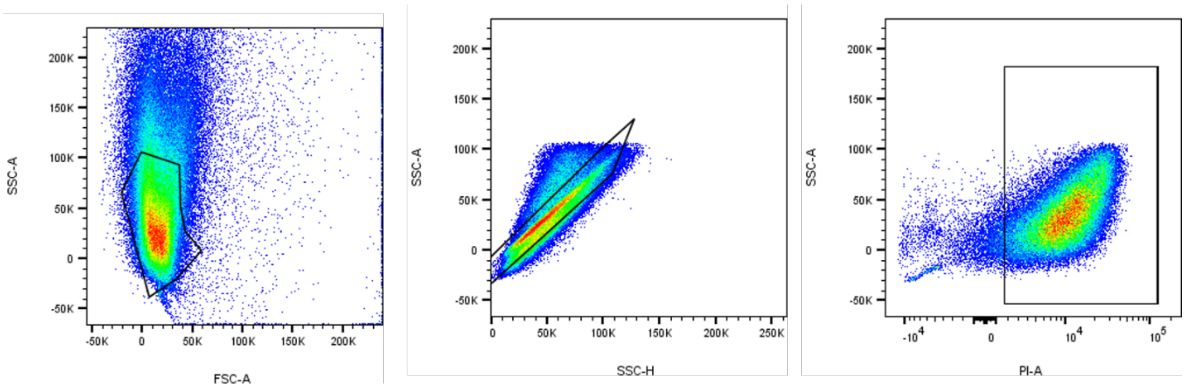

Supplementary Fig. 23 Gating strategy for bacterial Flow Cytometry.

	MB NPs	LPS-binding peptides	Metal nanomaterials	2D antibacterial materials
Materials	Nano-MgB_2<br>Nano-AlB_2<br>Nano-BeB_2	Cathelicidins	Silver<br>MgO<br>ZnO	BP<br>MoS_2<br>hBN
Structure	Hydrolytic 2D materials	Peptide	Low-dimensional materials	2D materials
Function	Anti-microbial<br>Anti-inflammation	Anti-microbial<br>Anti-inflammation	Anti-microbial	Anti-microbial
Mechanisms	(1)Trap LPS or PGN by forming stable borate ester bond<br>(2)Trap LPS or PGN to enhance the local cation concentration, leading to cell membrane damage<br>(3) Trap LPS or PGN to inhibit excessive inflammation	(1) Bind to LPS through electrostatic interaction, leading to cell membrane damage	ROS and Metal cation production	Cell membrane damage<br>Charge transfer<br>ROS production<br>Oxidative stress

Supplementary Table 1 The functional and mechanistic differences among MB NPs, LPS-binding peptides, Metal nanomaterials, and 2D antibacterial materials.

mGAPDH-F	CTTAGCCCCCCTGGCCAAG
mGAPDH-R	TGGTCATGAGCCCTTCCACA
mTNF-α-F	TCAAGGACTCAAATGGGCTTTC
mTNF-α-R	TGCAGAACTCAGGAATGGACAT
mIL-6-F	CTGCAAGAGACTTCCATCCAGTT
mIL-6-R	GGGAAGGCCGTGGTTGTC
mIL-1β-F	TAACCTGTGGCCTTGG
mIL-1β-R	TGTGCTCTGCTTGTGAG
mMCP-1-F	CAGGTCCCTGTCATGCTTCT

mMCP-1-R	GTGGGGCGTTAACTGCATCT
mHmox1-1-F	CAGAACCCAGTCTATGCCCC
mHmox1-1-R	GTGAGGCCCATACCAGAAGG

Supplementary Table 2 QPCR primers for mice.

Pseudomonas aeruginosa UCBPP-PA14 16S F (96bp)	TGCCTGGTAGTGGGGGATAA
Pseudomonas aeruginosa UCBPP-PA14 16S R(96bp)	TCTGATAGCGTGAGGTCCGA
gene-CIA_RS23525 F	GAAGAGCGGGAAATGTGGGA
gene-CIA_RS23525 R	GGGGAAGATCACATAGGGCG
gene-CIA_RS29995 F	ATCAGGAAGCCTGGCAGTTC
gene-CIA_RS29995 R	GCTTGAAGGTCACCACCTGT
gene-CIA_RS20225 F	AGTCCAAGGACGGCCTCTAT
gene-CIA_RS20225 R	CATCAGCCAGGGTATCGACC
gene-CIA_RS26230 F	ATCCACATCGACGGCATCAG
gene-CIA_RS26230 R	ATACGGTGCACGTAGTCGTC
gene-CIA_RS24915 F	AGATCGTCGACATCAAGGGC
gene-CIA_RS24915 R	CAGGATCACGTCCGCTTCAA
gene-CIA_RS33410 F	GACCGATCCGAAGGTCATCC
gene-CIA_RS33410 R	ATCGAGCGCACGTGGATATT
gene-CIA_RS35865 F	GTGATCGAGATCGCCGAAGT
gene-CIA_RS35865 R	GGTAGTCGATCAGGCGGATG
gene-CIA_RS36040 F	GCGACTTCACCATCAATGCC
gene-CIA_RS36040 R	CGATTGCGGATGTCGTGAAC
gene-CIA_RS20885 F	CACAGATCCTCGTCGCTACC
gene-CIA_RS20885 R	TTCCAGGTCGTCCATGAAGC
gene-CIA_RS33425 F	TCTTACGATTCTCGCCACGG
gene-CIA_RS33425 R	TTGATGTTGATGGCCGGGAA
gene-CIA_RS21575 F	CTGAACTTCATCCGCCAGGT
gene-CIA_RS21575 R	CTGGAAGCGATTGAACAGCG
gene-CIA_RS34960 F	CTGGAGAAGATCCACGACCG
gene-CIA_RS34960 R	GAGGTGAAGTGGGTGTAGGC
gene-CIA_RS29525 F	ATCCATGGCAACAAGAGCCA
gene-CIA_RS29525 R	CATGGGGCAACTGGTCGATA
gene-CIA_RS35975 F	CATCAAGCAGGACCGCTACA
gene-CIA_RS35975 R	CAGGCCGAATACGTCCTTGA
gene-CIA_RS08900 F	GTTAAGTTCGGCGATTCCGC
gene-CIA_RS08900 R	ACGACTTGTCCAGAACCACG
gene-CIA_RS34920 F	AGGGCATTCGCTACAAGACC
gene-CIA_RS34920 R	GTCGAAGGACTCGATCTGGC

Supplementary Table 3 QPCR primers for *Pseudomonas aeruginosa.*
