# Supplementary material for: Reactive metal boride nanoparticles trap lipopolysaccharide and peptidoglycan for bacteria-infected wound healing
Source: Nat Commun. 2022 Nov 29;13:7353. doi: 10.1038/s41467-022-35050-6 (PMC9708144; doi:10.1038/s41467-022-35050-6)
Supplement: Supplementary file 2 — Reporting Summary [file 41467_2022_35050_MOESM2_ESM.pdf]

nature portfolio

Corresponding author(s): Wenbo Bu

Last updated by author(s): Nov 6, 2022

Reporting Summary

Nature Portfolio wishes to improve the reproducibility of the work that we publish. This form provides structure for consistency and transparency in reporting. For further information on Nature Portfolio policies, see our Editorial Policies and the Editorial Policy Checklist.

Statistics

For all statistical analyses, confirm that the following items are present in the figure legend, table legend, main text, or Methods section.

n/a	Confirmed	
☐	☒	The exact sample size (n) for each experimental group/condition, given as a discrete number and unit of measurement
☐	☒	A statement on whether measurements were taken from distinct samples or whether the same sample was measured repeatedly
☐	☒	The statistical test(s) used AND whether they are one- or two-sided<br>*Only common tests should be described solely by name; describe more complex techniques in the Methods section.*
☐	☒	A description of all covariates tested
☐	☒	A description of any assumptions or corrections, such as tests of normality and adjustment for multiple comparisons
☐	☒	A full description of the statistical parameters including central tendency (e.g. means) or other basic estimates (e.g. regression coefficient) AND variation (e.g. standard deviation) or associated estimates of uncertainty (e.g. confidence intervals)
☐	☒	For null hypothesis testing, the test statistic (e.g. F, t, r) with confidence intervals, effect sizes, degrees of freedom and P value noted<br>*Give P values as exact values whenever suitable.*
☒	☐	For Bayesian analysis, information on the choice of priors and Markov chain Monte Carlo settings
☐	☒	For hierarchical and complex designs, identification of the appropriate level for tests and full reporting of outcomes
☒	☐	Estimates of effect sizes (e.g. Cohen's d, Pearson's r), indicating how they were calculated

Our web collection on statistics for biologists contains articles on many of the points above.

Software and code

Policy information about availability of computer code

Data collection	CytExpert software 2.4, Nikon A1 software 5.21.03, and 3DHISTECH CaseViewer 2.4.
Data analysis	GraphPad Prism 8.0.2, Flowjo_V10_CL,Origin 8.5,DigitalMicrograph 3.7,Jade 6.5,Avantage 5.979, clusterprofiler version 3.16.1

For manuscripts utilizing custom algorithms or software that are central to the research but not yet described in published literature, software must be made available to editors and reviewers. We strongly encourage code deposition in a community repository (e.g. GitHub). See the Nature Portfolio guidelines for submitting code & software for further information.

Data

Policy information about availability of data

All manuscripts must include a data availability statement. This statement should provide the following information, where applicable:

- Accession codes, unique identifiers, or web links for publicly available datasets
- A description of any restrictions on data availability
- For clinical datasets or third party data, please ensure that the statement adheres to our policy

The main data supporting the results in this study are available within the paper and its Supplementary Information. All data generated in this study are available from the corresponding authors.

Field-specific reporting

Please select the one below that is the best fit for your research. If you are not sure, read the appropriate sections before making your selection.

- [x] Life sciences
- [ ] Behavioural & social sciences
- [ ] Ecological, evolutionary & environmental sciences

For a reference copy of the document with all sections, see nature.com/documents/nr-reporting-summary-flat.pdf

Life sciences study design

All studies must disclose on these points even when the disclosure is negative.

Sample size	Sample sizes were determined on the basis of prior experimental work or a pilot study. No statistical calculations were made to determine sample size.
Data exclusions	All exclusion criteria were pre-established. For the in vivo studies, the criteria for exclusion were failure in administration, mistakes in tissue dissections, and health concerns unrelated to the procedure requiring euthanasia and exclusion.
Replication	All experiments were carried out with at least 3 replicate samples for each experimental group. All data are representative of at least three independent experiments with similar results, which was described in corresponding figure legends.
Randomization	Samples were allocated into experimental groups at random.
Blinding	The investigator was blinded to the group allocation during data collection such as in Flow-cytometry, Westernblot, Immuno-fluorescences, H&E staining and bacterial-killing experiments. For other experiments,blinding was not possible since the primary investigators performed the experiments from the beginning to the end due to the technical nature of the experiments.

Reporting for specific materials, systems and methods

We require information from authors about some types of materials, experimental systems and methods used in many studies. Here, indicate whether each material, system or method listed is relevant to your study. If you are not sure if a list item applies to your research, read the appropriate section before selecting a response.

Materials & experimental systems

n/a	Involved in the study	
	x	Antibodies
	x	Eukaryotic cell lines
x		Palaeontology and archaeology
	x	Animals and other organisms
x		Human research participants
x		Clinical data
x		Dual use research of concern

Methods

n/a	Involved in the study	
x		ChIP-seq
	x	Flow cytometry
x		MRI-based neuroimaging

Antibodies

Antibodies used	1. MPO (Abcam, ab208670, Rabbit monoclonal, EPR20257,1:100) ;<br>2. mF4/80 (Santacruz , sc-5266, Rat monoclonal antibody, BM8,1:50-1:500);<br>3.Donkey anti-Rabbit IgG (H+L) Highly Cross-Adsorbed Secondary Antibody, Alexa Fluor™ 594, ThermoFisher scientific, A-21207, 1:500;<br>4.Donkey anti-Rat IgG (H+L) Highly Cross-Adsorbed Secondary Antibody, Alexa Fluor™ 488, ThermoFisher scientific, A-21208, 1:500<br>5.P38 antibody (Abways, Cat:CY5488, Rabbit IgG, 9E1,1:1000);<br>6.Pp38 antibody (Abways, Cat:CY6391, Rabbit IgG, rabbit polyclonal antibody,1:1000);<br>7.ERK1/2 antibody (Abways, Cat:CY5487, Rabbit IgG, 9D12,1:1000);<br>8.pErk1/2 antibody (Abways, Cat:CY5277, Rabbit IgG, 5B7,1:1000);<br>9.JNK1/2/3 antibody (Abways, Cat:CY5490, Rabbit IgG, 9E3,1:1000);<br>10.pJNK1/2/3 antibody (Abways, Cat:CY5541, Rabbit IgG, 11C3,1:1000);<br>11.GAPDH (Abways, Cat:AB0037, Rabbit IgGI, 11B7,1:5000);
Validation	1.MPO:https://www.abcam.com/myeloperoxidase-antibody-epr20257-ab208670.html<br>2.F4/80:https://www.scbt.com/p/f4-80-antibody-bm8?requestFrom=search<br>3. Donkey anti-Rabbit IgG (H+L) https://www.thermofisher.cn/cn/zh/antibody/product/Donkey-anti-Rabbit-IgG-H-L-Highly-Cross-Adsorbed-Secondary-Antibody-Polyclonal/A-21207<br>4.Donkey anti-Rat IgG (H+L) https://www.thermofisher.cn/cn/zh/antibody/product/Donkey-anti-Rat-IgG-H-L-Highly-Cross-Adsorbed-

Secondary-Antibody-Polyclonal/A-21208
5.p38:http://abways.com/showproduct.asp?cid=CY5488 p38 MAPK Antibody
6.Pp38:http://abways.com/showproduct.asp?cid=CY6391 Phospho-p38 MAPK (Thr180/Tyr182) Antibody
7.ERK:http://abways.com/showproduct.asp?cid=CY5487 ERK1/2 Antibody
8.PErk:http://abways.com/showproduct.asp?cid=CY5277 Phospho-Erk1 (T202/Y204) + Erk2 (T185/Y187) Antibody
9.JNK :http://abways.com/showproduct.asp?cid=CY5490 JNK1/2/3 Antibody
10.PJNK:http://abways.com/showproduct.asp?cid=CY5541 Phospho-JNK1/2/3 (T183+T183+T221) Antibody
11.GAPDH:http://abways.com/showproduct.asp?cid=AB0037 GAPDH Antibody

Eukaryotic cell lines

Policy information about cell lines

Cell line source(s)	HaCaT and Raw264.7 cell lines were purchased from Procell Life Science&technology Co.,Ltd
Authentication	The cell lines were authenticated by the company using STR profiling.
Mycoplasma contamination	All the cells were tested and were not contaminated by mycoplasma.
Commonly misidentified lines (See ICLAC register)	No commonly misidentified cell lines were used.

Animals and other organisms

Policy information about studies involving animals; ARRIVE guidelines recommended for reporting animal research

Laboratory animals	Balb/c mice (7-8 weeks, male) were purchased from Charles River (CHINA Inc). House condition information was provided in the manuscript.
Wild animals	The study did not involve wild animals.
Field-collected samples	The study did not involve samples collected from the field.
Ethics oversight	The protocol was approved by East China Normal University Animal Care and Use Committee.

Note that full information on the approval of the study protocol must also be provided in the manuscript.

Flow Cytometry

Plots

Confirm that:

- [x] The axis labels state the marker and fluorochrome used (e.g. CD4-FITC).
- [x] The axis scales are clearly visible. Include numbers along axes only for bottom left plot of group (a 'group' is an analysis of identical markers).
- [x] All plots are contour plots with outliers or pseudocolor plots.
- [x] A numerical value for number of cells or percentage (with statistics) is provided.

Methodology

Sample preparation	1.For the live and dead bacteria detection, P. aeruginosa (108/mL) treated with 272 μM Nano-MgB2 (12.5 μg/mL) and 544 μM H3BO3+Mg2+ for 3 h were added with 15 μM Propidium iodide and incubated for 30 min in the dark.For S. aureus, S. aureus (1×108/mL) were incubated with 10.88mM Nano-MgB2 (500 μg/mL) or 21.76 mM H3BO3+Mg2+ for 3h and then incubated with 15 μM Propidium iodide for 30 min in the dark.<br>2.For the bacteria cytoplasmic membrane depolarization assay , bacteria were suspended by a final concentration of 2 μM DiSC3(5) and incubated for 30 min. The change in fluorescence was measured immediately after the addition of 272 μM Nano-MgB2 and 544 μM H3BO3+Mg2+.
Instrument	FACScan (BD LSRFortessa X-20),FACScan (Beckman Coulter)
Software	Data were collected with CytExpert software 2.4 and analyzed with FlowJo_V10 software.
Cell population abundance	100,000 bacteria were acquired from the first gate with gate strategy of Forward Scatter Area (FSA) and Side Scatter Area (SSA) and more than 95% were involved in the following date analysis.
Gating strategy	1.Bacterial Live and dead gating strategy: Using Forward Scatter Area (FSA) and Side Scatter Area (SSA) to find the main group of bacteria; Using SSA and Side Scatter SSH(Side Scatter High) to remove adherent bacterial populations; Using SSH and PE to display the positive cell population of dead bacteria.

2.Bacterial cytoplasmic membrane depolarization assay gating strategy: Using Forward Scatter Area (FSA) and Side Scatter Area (SSA) to find the main group of bacteria; Using SSA and Side Scatter SSH(Side Scatter High) to remove adherent bacterial populations, bacteria cell membrane potential was shown in Histogram form.

☒ Tick this box to confirm that a figure exemplifying the gating strategy is provided in the Supplementary Information.
