# Supplementary figures and images for: Reactive metal boride nanoparticles trap lipopolysaccharide and peptidoglycan for bacteria-infected wound healing
Source: Nat Commun. 2022 Nov 29;13:7353. doi: 10.1038/s41467-022-35050-6 (PMC9708144; doi:10.1038/s41467-022-35050-6)

GAPDH

35  
25

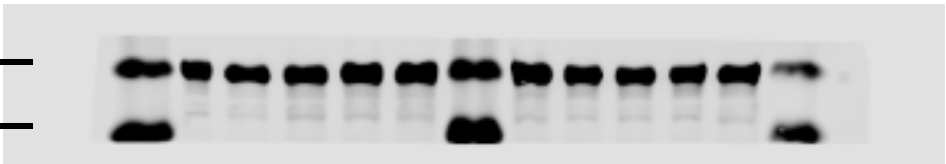

ERK

40  
35

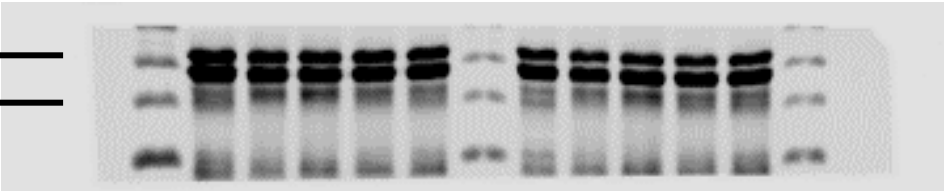

pERK

40  
35

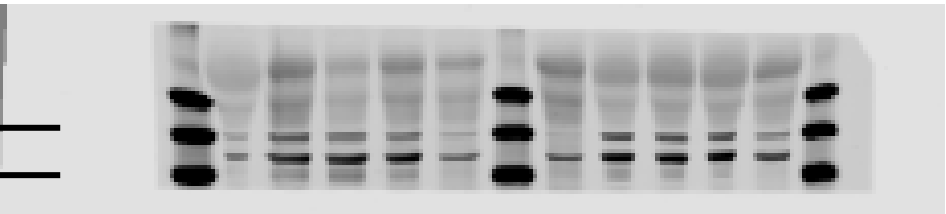

P38

40  
35

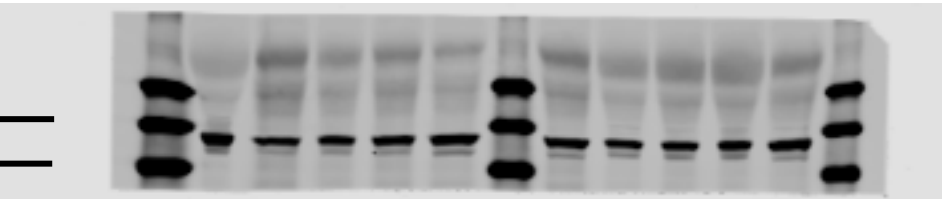

pP38

55  
40  
35

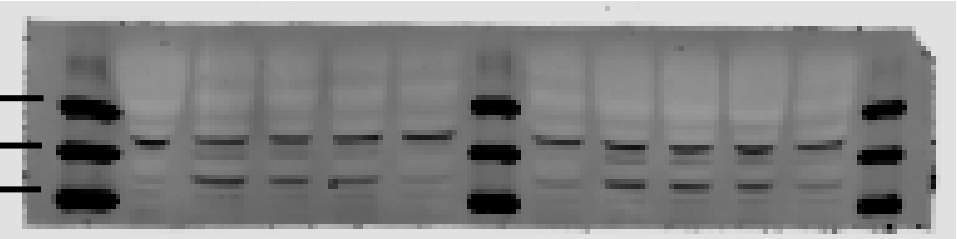

JNK

55  
40

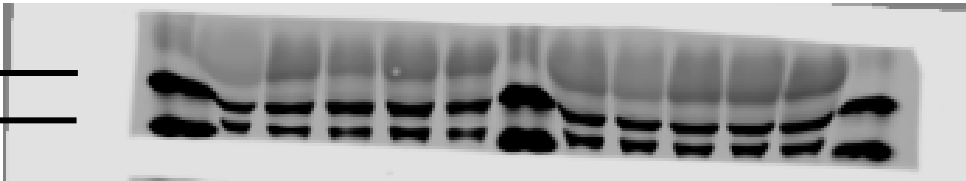

pJNK

55  
40

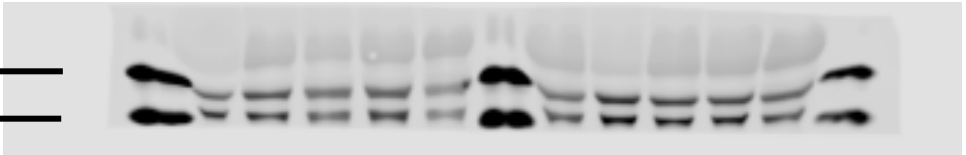

Supplement: Supplementary file 3 — Source Data [file 41467_2022_35050_MOESM3_ESM.zip › Source Data/source Data File 2.pdf]
